# Gene Expression Profiling in Slow-Type Calf *Soleus* Muscle of 30 Days Space-Flown Mice

**DOI:** 10.1371/journal.pone.0169314

**Published:** 2017-01-11

**Authors:** Guido Gambara, Michele Salanova, Stefano Ciciliot, Sandra Furlan, Martina Gutsmann, Gudrun Schiffl, Ute Ungethuem, Pompeo Volpe, Hanns-Christian Gunga, Dieter Blottner

**Affiliations:** 1 Center for Space Medicine Berlin, Neuromuscular Group, Charité Universitätsmedizin Berlin, Berlin, Germany; 2 Vegetative Anatomy, Charité Universitätsmedizin Berlin, Berlin, Germany; 3 Venetian Institute of Molecular Medicine, Padova, Italy; 4 Department of Medicine (DIMED), University of Padova, Padova, Italy; 5 Institute of Neuroscience Consiglio Nazionale delle Ricerche, Padova, Italy; 6 Laboratory of Functional Genomics, Charité Universitätsmedizin Berlin, Berlin, Germany; 7 Centre for Space Medicine, Department for Physiology, Charité Universitätsmedizin Berlin, Berlin, Germany; University of Valencia, SPAIN

## Abstract

Microgravity exposure as well as chronic disuse are two main causes of skeletal muscle atrophy in animals and humans. The antigravity calf *soleus* is a reference postural muscle to investigate the mechanism of disuse-induced maladaptation and plasticity of human and rodent (rats or mice) skeletal musculature. Here, we report microgravity-induced global gene expression changes in space-flown mouse skeletal muscle and the identification of yet unknown disuse susceptible transcripts found in *soleus* (a mainly slow phenotype) but not in *extensor digitorum longus* (a mainly fast phenotype dorsiflexor as functional counterpart to *soleus*). Adult C57Bl/N6 male mice (n = 5) flew aboard a biosatellite for 30 days on orbit (BION-M1 mission, 2013), a sex and age-matched cohort were housed in standard vivarium cages (n = 5), or in a replicate flight habitat as ground control (n = 5). Next to disuse atrophy signs (reduced size and myofiber phenotype I to II type shift) as much as 680 differentially expressed genes were found in the space-flown *soleus*, and only 72 in *extensor digitorum longus* (only 24 genes in common) compared to ground controls. Altered expression of gene transcripts matched key biological processes (contractile machinery, calcium homeostasis, muscle development, cell metabolism, inflammatory and oxidative stress response). Some transcripts (Fzd9, Casq2, Kcnma1, Ppara, Myf6) were further validated by quantitative real-time PCR (qRT-PCR). Besides previous reports on other leg muscle types we put forth for the first time a complete set of microgravity susceptible gene transcripts in *soleus* of mice as promising new biomarkers or targets for optimization of physical countermeasures and rehabilitation protocols to overcome disuse atrophy conditions in different clinical settings, rehabilitation and spaceflight.

## Introduction

Long-term exposure to microgravity (μG) or extended periods of disuse are two main causes of the reduction of skeletal muscle mass and performance in animals and humans in space. The understanding of the molecular mechanism underlying this process is crucial for the identification of efficient countermeasures able to ameliorate or even completely prevent disuse-induced skeletal muscle maladaptation. Due to the limited number of spaceflights, the first challenge in space biomedical research is the availability of large cohorts of biological samples (tissues, cells, body fluids, etc.) exposed to microgravity. In this regard, the use of small rodents, in particular mice, represent a suitable model for studying the effects of microgravity *in vivo*. The mouse genome has many similarities with human genome and it can be easily engineered in order to generate pathological models. Although the estimated life span of mice is much shorter (approx. 1.5 to 2 yrs.) compared to humans (approx. 76 to82 yrs., industrialized countries), the results obtained from murine animals in short or mid-duration spaceflight (7 to 30 days) might be comparable to longer periods of microgravity exposure (up to six months or even more) in humans.

In order to better understand the molecular mechanism of disuse and microgravity-induced skeletal muscle atrophy, a more comprehensive analysis of the gene expression maladaptation in different regional and functional muscle types of vertebrates (including human) is urgently needed. Previous studies performed in space-flown rodents (mainly rats) showed that microgravity exposure mainly affected lower limb calf muscles responsible for the antigravity response required for standing tasks, movement or physical performance on Earth [1]. The major effects of microgravity on the structure and function of animal and human skeletal muscle were previously reported [2–9]. Many of the morphological and biochemical changes observed in real microgravity were, at least in part, also found in laboratory animal models (such as rat and mouse hind limb unloading / tail-suspension) of disuse [10, 11] as well as in humans using bed rest as analog to spaceflight [12, 13].

However, few studies focused on the global gene expression changes induced by spaceflight. For example, changes in gene expression were mainly reported in the mouse and rat gastrocnemius following 11, 16 or 17 days space flights [14–16]. In particular, Taylor W.E. and co-worker identified genes involved in the control of cell proliferation (p21, Rb), cell cycle (Cyclins) and signaling pathways (MAPK3, RAB2) differentially expressed in *gastrocnemius* and *tibialis anterior* of rats flown onboard of the NASA STS-90 Neurolab spaceflight for 17 days [16]. A complete study by Nikawa T. and co-workers compared the global gene expression among spaceflight, tail-suspended and denervated rat *gastrocnemius*, concluding that genes altered specifically in microgravity condition were linked to cytoskeletal molecules and ubiquitin ligase genes [15]. More recently, Allen DL and co-worker investigated by means of Affymetrix microarray the gene expression profile in *gastrocnemius* of mice flown onboard of the STS-108 space shuttle flight for as short as 11 days on orbit. They compared the resulting gene expression profile with the transcriptome of tail suspended mice without and with reloading (3.5 h), showing that the expression of a high number of genes differentially regulated following microgravity exposure was also altered in the unloading condition and that the short reloading counteracted the effect of weightlessness for the majority of the identified genes [14]. In a long-duration space experiment (>90 days), Sandona and co-workers analyzed by quantitative real time PCR the changes in the expression of 29 genes in mouse *soleus* and EDL compared to ground controls. MuRF-1 transcript and different protein kinase C isoforms were found upregulated mainly in soleus of the space-flown mouse, while transcripts linked to stress related genes were found upregulated mainly in EDL [7]. However, the global gene expression profile of the slow-type *soleus* muscle following microgravity exposure is still missing. Recently, two studies showed mainly structural and biochemical changes found in the *gastrocnemius*, *soleus* and *anterior tibialis* of space-flown mice on board of the BION-M1 [17–19].

The present study aims to systematically analyze for the first time by means of microarray technology the global gene expression changes in *soleus* muscle of mice following 30 days of spaceflight. The calf *soleus* is one of the most studied reference postural hind limb muscle in rodents and humans to investigate skeletal muscle adaptation and plasticity changes following periods of disuse in clinical research [20] and aging.

In the present experimental setting, adult male C57BL/N6 mice were either flown aboard the BION-M1 biosatellite for 30 days on orbit (BION-flown = BF) or housed as sex- and age-matched cohorts (n = 5 each) in a replicate flight habitat on Earth (BION-ground = BG) as reference flight control, and in standard animal cages concomitant to the duration of the biosatellite flight (flight control = FC). Mice flown aboard of the biosatellite were in good condition after landing, as previously reported by Andreev-Andrievskiy [17]. More in detail, the climate parameters in flight and ground controls were within the range suitable for rodents’ housing and no significant differences in the bodyweight of mice of the different experimental groups were found [17]. As previously reported the mean weight of the *soleus* muscle was significantly reduced (21.9%) in BF group (p < 0.05) compared with FC [18].

Besides the known microgravity transcriptional changes previously studied in space-flown fast type muscle, we now highlighted a large number of yet unidentified microgravity-sensitive transcripts in the slow-type *soleus* (out of 680 genes in total) of space-flown mice (BF vs. BG) compared to much smaller amounts of altered transcripts found in space-flown fast-type EDL (72 genes, with only 24 genes in common with *soleus*). The large scale gene expression analysis approach used in the present work provides a more specific and comprehensive data set obtained from two very different functional muscle types (*soleus* vs EDL) in long-duration (30 days) space-flown mice. In conclusion, this study contributes to expand the basic knowledge on gene expression adaptation involved in microgravity-induced skeletal muscle atrophy in mouse *soleus*, and to highlight potential new biomarkers or targets for the improvement of countermeasures able to ameliorate or even prevent disuse atrophy in rehabilitation, different clinical settings and in spaceflight.

## Materials and Methods

### Ethical approval

All animal procedures were performed according to the European Convention for the Protection of Vertebrate Animals used for Experimental and Other Scientific Purposes (Strasbourg, 18.03. 1986). The study was approved by Institutional Animal Care and Use Committee (IACUC) of MSU Institute of Mitoengineering (Protocol 35, November 1, 2012) and by Biomedical Ethics Commission of Institute for Biomedical Problems (IBMP), Moscow (protocol 319, April 4, 2013).

### Animals

C57BL/N6 mice (22–25 g) were purchased from the Animal Breeding Facility Branch of Shemyakin & Ovchinnikov Institute of Bioorganic Chemistry, Russia. Mice were transported to the animal facility of Moscow State University, Institute of Mitoengineering, for preadaptation training and selection in the laboratory setting on the ground before flight. For all experiments, 19–20 weeks old male mice were used. In detail, following a preflight animal training and pre-selection program (e.g., pre-adaptation to standard laboratory cage conditions and familiarization of individual mice groups compliant for housing in smaller animal flight habitats used for spaceflight mission as previously proposed [21]), mice were randomly divided in 3 groups: mice to be flown aboard the BION-M1 biosatellite exposed for 30 days to microgravity (BION Flown = BF), mice housed for 30 days under the same biosatellite bio-parameters (i.e., number of animals per group and identical housing conditions in a BION-M1 used habitat) on ground (BION Ground = BG), and mice housed in the animal facility of Moscow State University (Institute of Mitoengineering) concomitant to the duration of the biosatellite flight (FC = Flight Control). Before launch to space, mice were pre-adapted for 2 weeks on the ground to a special diet later provided in their space habitats. Thus, BF (flown mice) and BG (ground control mice) mice were fed with “space” paste food including all necessary major nutrients comparable to standard chow and water developed by the Institute for Biomedical Problems (IBMP, Moscow), while FC mice were fed with standard chow and water *ab libitum*. Food and water intake within the different experimental groups were previously reported by Andreev-Andrievskiy and co-workers [17].

### Sample preparation and transportation

After landing, mice were transferred in the BION-M1 housing device within 12–14 hrs. from the landing site (Kazakhstan) to Moscow Institute of Biomedical Problems (IMBP), Russia. Operational support with preflight animal handling, post flight animal tissue dissection and sample freezing was accomplished by our Russian contracted partners on site (IMBP, Moscow, Russia, contract # BION-M1/2013 between RF SRC-IMBP and Charité Berlin, Germany). All samples were delivered deeply frozen on dry ice from IMBP Moscow via temperature controlled express delivery parcels (World Courier Express) to the Charité Berlin, Germany, and further processed in our laboratory.

### Immunohistochemistry and morphological analysis

Mice muscle cryosections (8 μm thickness) from *soleus* (n = 2) and EDL (n = 2) were cut with a cryostat (CM 1850, Leica Microsystems, Bensheim, Germany), mounted on charged slides, stored frozen in sealed plastic boxes (minus 80°C), and either standard hematoxylin-eosin (H&E) stained for overview, and triple-immunolabeled with the following anti-MyHC isoform monoclonal antibodies as already described (8): BA-D5 that recognizes type 1 MyHC isoform; SC-71 for type 2A MyHC isoform; BF-F3, for type 2B MyHC isoform (DSHB, Developmental Studies Hybridoma Bank, University of Iowa, Iowa City, IA). The sections were co-stained with an anti-dystrophin antibody (Santa Cruz Biotechnology, Santa Cruz, CA) to allow measurement of fiber size. In all protocols, we used goat anti rabbit Alexa-635 conjugated secondary antibody for dystrophin staining and goat anti-mouse Alexa-555, goat anti-mouse Alexa-488 and goat anti-mouse Alexa 405-conjugated secondary antibody diluted to a final concentration of 1 μg/ml for myosin heavy chain (MyHC) antigens. For mouse derivatives of monoclonal primary and secondary antibodies, the MOM Ig blocking reagent (Vector Laboratories, Burlingame, CA, USA) was used to block mouse IgG background. Immunostained sections were analyzed with an epifluorescence microscope (Axioplan; Zeiss, Oberkochen, Germany) equipped with a Cool Snap digital camera (Visitron Systems GmbH, Puchheim, Germany). Digitized images were processed with MetaVue software (Meta Series 7.5.6; system ID: 33693; Molecular Devices, Sunnyvale, CA, USA). To assess the cross-sectional area (CSA) of the different myofiber types, digitized photographs were acquired and the myofiber CSA was automatically measured by means of ImageJ 1.45 g (NIH, freeware imaging software).

### RNA extraction and sample target preparation

Total RNA was isolated from mouse *soleus* (n = 3) and EDL (n = 3) muscles of each experimental group (BF, BG and FC) using the acid guanidinium thiocyanate-phenol-chloroform extraction followed by silica-membrane purification. Frozen tissue samples were ground to a fine powder under liquid nitrogen. A homogeneous lysate was achieved by adding lysing buffer and flowing it 10 times through a needle of a sterile syringe. Tissue lysate was centrifuged and the supernatant was used for RNA phenol/chloroform extraction. After phase separation, the aqueous layer was transferred and mixed with an equal volume of 70% ethanol. Then, the total RNA was extracted using RNeasy spin columns RNeasy micro Kit (Qiagen, Hilden, Germany) according to the manufacturer’s protocol. 2100 Bioanalyzer (Agilent technologies, PA, USA) was used to check RNA integrity. The amplification and labeling of the RNA samples were carried out according to the manufacturer's instructions (Affymetrix, Santa Clara, CA). Briefly, total RNA was quantified by and checked by analysis on a LabChip (BioAnalyzer, AGILENT Technologies, Santa Clara, CA). The GeneChip® 3’ IVT Express Protocol is based on the Eberwine or reverse transcription method (in vitro transcription, IVT). Starting from 100 nanogram total RNA, first strand DNA was synthesized, containing a T7 promotor sequence and then converted into a double-stranded DNA. The double strand DNA serves as template in the subsequent in vitro transcription (IVT) reaction. This amplification step generates biotin labeled complementary RNA (cRNA). After cleanup the biotin-modified RNA was fragmented by alkaline treatment. 15 μg of each cRNA sample was hybridized for 16 hours at 45°C to an Affymetrix GeneChip Mouse 430A 2.0 Array. Arrays were washed and stained with streptavidin-phycoerythrin solutions using a fluidics station according to the protocols recommended by the manufacturer. Finally, probe arrays were scanned at 1.56-μm resolution using the Affymetrix GeneChip System confocal scanner 3000. Affymetrix Mouse Genome 430A 2.0 Array includes 22 600 probes sets to evaluate the expression level of more than 14000 well characterized mouse genes.

### Microarray data analysis and pathway analysis

Data analysis was performed importing.cel files to the Partek^®^ Genomics Suite^®^ 6.6 software. Robust Multichip Average algorithm (RMA) was used for data normalization. The processing steps involved in the RMA method are: background correction, quantile probe normalization across all arrays of the experiments, Log2 transformation of all signal values and median polished Probe set summarization. To test for differences in means between groups Analysis of Variance (ANOVA) with one grouping variable (one–way ANOVA) was applied [22]. Genes that have any changes with p value lower than 0.05 and fold changes >2 or <-2 were assumed / defined to be differentially regulated. In the analysis of genes differentially regulated in *soleus* muscle a step up false discovery rate (FDR) [23] was applied to p values from linear contrasts to determine a cut-off for significantly differentially expressed genes. Lists comprised genes with fold changes more than 2 or less than -2. Annotation was performed according to the Mouse Genome 430A 2.0 Array Probe Set Annotations. Microarray data were deposited in Gene Expression Omnibus (GEO) repository accession number: GSE80223. Principal Component Analysis (PCA) was used as exploratory technique to reduce the dimensionality of these high dimensional data [24]. It is based on a linear transformation and preserves the variation in the data. The PCA analysis was configured as followed: As dispersion matrix the covariance method was chosen and during computation of the covariance matrix, the data was mean-centred. The eigenvectors were scaled using the normalization mode (orthogonal and scaled to unity), as described in Partek Genomic Suite 6.6 user manual.

To group the genes of interest (680 regulated genes in *soleus* BF vs. BG) into biological processes, a Gene ontology analysis was performed by using GO enrichment tool of Partek^®^ Genomics Suite^®^ software. The Fisher's Exact test on the counts of genes was used to identify interesting functional groups and pathways with respect to an enrichment p value < 0.05 and more than 8 genes per group. Further gene ontology and pathway analysis were performed using the Functional Annotation Clustering module of DAVID v6.7 (The Database for Annotation, Visualization and Integrated Discovery). To evaluate the significance on gene enrichment a modified Fisher’s exact (EASE score < 0.05) test has been applied [25] and the resulting genes linked to the identified functional gene clusters were included in Tables 1 and 2, S1, S2 and S3 Tables. Pathway enrichment analysis was performed be means of KEGG pathway module of WEB-based GEne SeT AnaLysis Toolkit [26] focusing only on genes significantly differentially regulated in *soleus* and EDL of BF vs. BG. For this analysis p value from hypergeometric test and p value adjusted by the multiple test adjustment were calculated and included in the corresponding table.

**Table 1:** Functional gene clusters differentially regulated in *soleus* of BION-M1 space flown mice.

					SOL						EDL			
			BF vs. BG		FC vs. BG		BF vs. FC		BF vs. BG		FC vs. BG		BF vs. FC	
	Entrez Gene	Gene Symbol	p-value	*FC*	p-value	*FC*	p-value	*FC*	p-value	*FC*	p-value	*FC*	p-value	*FC*
**contractile fiber**	11474	**Actn3**	7,48E-06	**21,58**	0,20284	**1,74**	5,12E-05	**12,41**	0,98737	**-1,01**	0,945965	**1,03**	0,933378	**-1,04**
	17884	**Myh4**	0,0007	**19,20**	0,79069	**1,19**	0,00113	**16,08**	0,93138	**1,06**	0,87741	**1,11**	0,945627	**-1,05**
**glucose metabolic process**	14120	**Fbp2**	0,00035	**4,15**	0,33063	**1,34**	0,00204	**3,10**	0,60133	**-1,17**	0,906368	**1,04**	0,523732	**-1,21**
	72141	**Adpgk**	4,17E-05	**3,97**	0,88647	**1,03**	5,20E-05	**3,84**	0,90908	**1,03**	0,662455	**1,10**	0,746421	**-1,08**
**fatty acid metabolism**	113868	**Acaa1a**	0,00222	**-2,11**	0,49962	**1,14**	0,00065	**-2,41**	0,55026	**1,13**	0,026848	**1,63**	0,080798	**-1,44**
	12896	**Cpt2**	5,19E-05	**-2,32**	0,19581	**1,21**	7,35E-06	**-2,81**	0,68653	**-1,06**	0,479292	**1,11**	0,275055	**-1,17**
**regulation of lipid metabolic process**	11606	**Agt**	3,10E-06	**3,68**	0,59576	**-1,09**	1,58E-06	**4,02**	0,2658	**1,21**	0,41027	**1,15**	0,758884	**1,05**
	20411	**Sorbs1**	0,00888	**-2,44**	0,2651	**-1,40**	0,07503	**-1,75**	0,02916	**2,03**	0,096792	**1,67**	0,512651	**1,21**
**inflammatory response**	20293	**Ccl12**	0,00039	**6,42**	0,72736	**1,15**	0,00071	**5,60**	0,56139	**1,26**	0,737582	**1,14**	0,803498	**1,10**
	20296	**Ccl2**	0,00136	**5,14**	0,57028	**-1,26**	0,00049	**6,48**	0,6249	**-1,22**	0,68152	**-1,18**	0,936598	**-1,03**
	20306	**Ccl7**	0,00041	**2,79**	0,69608	**-1,09**	0,00021	**3,04**	0,4399	**1,19**	0,902712	**1,03**	0,513085	**1,15**
**cellular calcium ion homeostasis**	16438	**Itpr1**	0,00547	**2,63**	0,12286	**1,61**	0,11101	**1,64**	0,49471	**1,22**	0,159926	**1,53**	0,442663	**-1,25**
	18750	**Prkca**	7,98E-06	**2,01**	0,18989	**1,14**	5,87E-05	**1,76**	0,01436	**1,31**	0,402483	**1,09**	0,069658	**1,21**
**stress response**	81489	**Dnajb1**	0,00147	**10,16**	0,68665	**-1,26**	0,00071	**12,84**	0,79053	**-1,17**	0,579058	**-1,38**	0,770364	**1,18**
**regulation of muscle contraction**	11938	**Atp2a2**	0,00035	**-2,74**	0,77862	**1,06**	0,00022	**-2,91**	0,50012	**-1,15**	0,840265	**1,04**	0,385186	**-1,20**
	12373	**Casq2**	1,82E-07	**-4,16**	0,92487	**-1,01**	2,01E-07	**-4,11**	0,69434	**-1,06**	0,475631	**-1,10**	0,744269	**1,05**
**muscle organ development**	17927	**Myod1**	0,00432	**4,72**	0,3483	**1,54**	0,02633	**3,06**	0,45904	**-1,40**	0,981518	**1,01**	0,445604	**-1,42**
	17878	**Myf6**	4,25E-05	**2,58**	0,0477	**-1,40**	2,12E-06	**3,60**	0,00053	**2,03**	0,378428	**-1,15**	0,000117	**2,33**
**response to oxidative stress**	18104	**Nqo1**	0,00156	**-2,12**	0,40971	**-1,17**	0,00744	**-1,81**	0,61575	**1,10**	0,297234	**1,22**	0,576249	**-1,11**
	12359	**Cat**	0,00235	**-2,48**	0,15179	**-1,44**	0,03943	**-1,73**	0,32971	**1,27**	0,061393	**1,63**	0,315489	**-1,28**
**regulation of programmed cell death**	12575	**Cdkn1a**	0,0035	**2,61**	0,00424	**-2,54**	1,18E-05	**6,61**	0,00178	**2,88**	0,02099	**-2,02**	2,37E-05	**5,81**
	14311	**Cidec**	0,00072	**5,58**	0,24753	**1,59**	0,0065	**3,51**	0,16671	**1,75**	0,063929	**2,18**	0,580255	**-1,24**
**Focal adhesion**	21894	**Tln1**	0,00011	**2,45**	0,29812	**-1,19**	2,15E-05	**2,91**	0,61681	**-1,09**	0,56217	**-1,10**	0,935643	**1,01**
**Other/ unknown**	19017	**Ppargc1a**	0,00267	**-2,55**	0,05259	**-1,70**	0,13144	**-1,49**	0,39786	**-1,24**	0,56415	**1,16**	0,167349	**-1,44**
	170826	**Ppargc1b**	0,00023	**-2,22**	0,35479	**1,16**	5,07E-05	**-2,57**	0,12471	**-1,29**	0,771826	**-1,05**	0,200667	**-1,23**
	19013	**Ppara**	2,75E-05	**-4,18**	0,72112	**1,08**	1,63E-05	**-4,53**	0,0683	**-1,55**	0,874348	**-1,04**	0,090404	**-1,50**

The differentially regulated genes (BF vs. BG) in *soleus* meeting FDR < 0.05 and < -2 & > 2 fold change criteria were analysed by DAVID database and a short list of genes linked to the main functional clusters is included in this table, for the complete gene list see supporting information, S1, S2 and S3 Tables. Fold changes of the corresponding genes in EDL are shown only for comparison. Functional clusters with an EASE score < 0.05 were included in this table.

**Table 2:** Functional gene clusters differentially regulated in EDL of BION-M1 space flown mice.

					EDL						SOL			
			BF vs. BG		FC vs. BG		BF vs. FC		BF vs. BG		FC vs. BG		BF vs. FC	
	Entrez Gene	Gene Symbol	p-value	*FC*	p-value	*FC*	p-value	*FC*	p-value	*FC*	p-value	*FC*	p-value	*FC*
**intermediate filament**	56430	**Clip1**	0,029498	**2,13**	0,05286	**1,93**	0,752838	**1,10**	0,15357	**-1,60**	0,77867	**-1,09**	0,240127	**-1,46**
** **	16663	**Krt13**	0,031692	**-7,50**	0,06507	**-5,39**	0,69603	**-1,39**	0,939823	**-1,07**	0,800275	**1,24**	0,742852	**-1,32**
** **	16673	**Krt36**	0,046406	**-2,10**	0,12672	**-1,73**	0,573065	**-1,21**	0,938344	**-1,03**	0,82394	**-1,08**	0,884481	**1,05**
** **	16687	**Krt6a**	0,038246	**-3,77**	0,1034	**-2,73**	0,582534	**-1,38**	0,870124	**1,10**	0,59753	**1,36**	0,714003	**-1,24**
** **	17918	**Myo5a**	6,01E-07	**6,00**	0,28696	**1,23**	2,23E-06	**4,87**	1,10E-06	**5,43**	0,126039	**1,36**	8,75E-06	**3,99**
**response to peptide hormone stimulus**	13685	**Eif4ebp1**	3,45E-05	**2,30**	0,04117	**1,35**	0,00146	**1,71**	6,11E-05	**2,19**	0,9893	**1,00**	6,24E-05	**2,18**
** **	18708	**Pik3r1**	0,000323	**3,23**	0,58411	**1,14**	0,000847	**2,83**	0,0937057	**1,54**	0,375708	**-1,24**	0,017917	**1,91**
** **	20716	**Serpina3n**	0,000634	**2,21**	0,43748	**1,15**	0,002647	**1,92**	0,0025642	**1,93**	0,239112	**-1,24**	0,000294	**2,39**
** **	20411	**Sorbs1**	0,029162	**2,03**	0,09679	**1,67**	0,512651	**1,21**	0,0088839	**-2,44**	0,265102	**-1,40**	0,075028	**-1,75**
**acute inflammatory response**	11537	**Cfd**	0,017779	**2,42**	0,00226	**3,46**	0,286007	**-1,43**	0,003979	**3,13**	0,041254	**2,08**	0,229282	**1,50**
** **	18405	**Orm1**	0,019054	**2,22**	0,33444	**1,35**	0,114579	**1,65**	7,98E-05	**5,59**	0,649953	**1,15**	0,000167	**4,88**
**transcription factor activity**	13170	**Dbp**	0,029217	**-2,83**	0,06687	**2,33**	0,000739	**-6,62**	0,0076623	**-3,84**	0,009637	**3,65**	4,11E-05	**-14,01**
** **	17878	**Myf6**	0,000532	**2,03**	0,37843	**-1,15**	0,000117	**2,33**	4,25E-05	**2,58**	0,047703	**-1,40**	2,12E-06	**3,60**
** **	18029	**Nfic**	0,000693	**-2,02**	0,00039	**-2,13**	0,737947	**1,05**	0,0017416	**-1,86**	0,000612	**-2,04**	0,564467	**1,10**
** **	217166	**Nr1d1**	0,012745	**-2,50**	0,0707	**-1,86**	0,365406	**-1,34**	0,0647039	**1,89**	0,631823	**-1,17**	0,026645	**2,21**
** **	16658	**Mafb**	0,024137	**-2,37**	0,37981	**-1,36**	0,121324	**-1,75**	0,110666	**-1,78**	0,446732	**-1,30**	0,367918	**-1,37**
**Others/ unknown**	21928	**Tnfaip2**	0,00117	**-2,48**	0,24487	**1,30**	0,000147	**-3,22**	0,0329899	**-1,68**	0,059973	**1,56**	0,000745	**-2,61**
** **	14181	**Fgfbp1**	0,020565	**-2,03**	0,88068	**1,04**	0,015481	**-2,11**	0,328204	**1,31**	0,660076	**-1,13**	0,167245	**1,48**
** **	20878	**Aurka**	0,000537	**-2,31**	0,97911	**-1,00**	0,000562	**-2,30**	0,531265	**1,12**	0,518459	**-1,13**	0,214734	**1,26**
** **	29818	**Hspb7**	0,032126	**2,15**	0,02475	**-2,25**	0,000315	**4,82**	0,269494	**-1,44**	0,000506	**-4,41**	0,00398	**3,06**
** **	15926	**Idh1**	0,000172	**2,28**	0,83134	**1,03**	0,000246	**2,21**	0,646801	**-1,08**	0,384587	**-1,15**	0,673081	**1,07**
** **	16194	**Il6ra**	0,000585	**2,30**	0,124	**1,35**	0,011677	**1,71**	0,003787	**1,91**	0,003074	**1,95**	0,911215	**-1,02**
** **	12575	**Cdkn1a**	0,004445	**2,60**	0,04179	**-1,87**	8,87E-05	**4,86**	0,0063612	**2,47**	0,045838	**-1,84**	0,000131	**4,54**
** **	11433	**Acp5**	0,019126	**2,73**	0,33396	**1,45**	0,115174	**1,88**	0,0064448	**3,39**	0,359508	**1,42**	0,037491	**2,38**
** **	56349	**Net1**	0,000327	**2,96**	0,11008	**-1,46**	2,22E-05	**4,32**	0,0217705	**1,78**	0,002552	**-2,29**	3,25E-05	**4,07**
** **	68916	**Cdkal1**	0,000994	**3,11**	0,62588	**1,14**	0,002437	**2,73**	0,0009238	**3,15**	0,92231	**1,03**	0,001101	**3,07**
** **	18948	**Pnmt**	6,55E-05	**3,53**	0,47128	**-1,17**	2,16E-05	**4,14**	0,004559	**2,09**	0,732886	**-1,08**	0,002405	**2,25**

The differentially regulated genes (BF vs. BG) in EDL meeting p value < 0.05 (2 way ANOVA) and < -2 & > 2 fold change criteria were analysed by DAVID database and included in this table. Fold changes of the corresponding genes in *soleus* are shown only for comparison. Functional clusters with an EASE score < 0.05 were included in this table.

### Quantitative PCR validation

Quantitative PCR was performed by the SYBR Green method as previously described [27]. Briefly, 400 ng of RNA were converted to cDNA by using random hexamers and SuperScript^®^ VILO™ (Invitrogen) following the manufacturer’s instructions. Specific primers for qPCR were already published [7] or designed using Primer3 software (http://frodo.wi.mit.edu/, Whitehead Institute for Biomedical Research). Their thermodynamic specificity was determined using BLAST sequence alignment (NCBI) and vector NTI^®^ software (Invitrogen). Oligonucleotide primers used are listed in S4 Table. The reaction mix consists of 10 μl of 2x iQ SYBR Green Supermix (Bio-Rad), 0.3 pmol/μl primers, 8 ng of cDNA and DNase/RNase-free water up to 20 μl. The PCR parameters were initial denaturation at 95°C for 30 s followed by 40 cycles of 10 s at 95°C and 30 s at the corresponding annealing temperature (55–59°C) for acquisition of fluorescence signal. A melting curve was generated by the iQ5 software (Biorad) following the end of the final cycle for each sample, by continuous monitoring the SYBR Green fluorescence throughout the temperature ramp from 65°C to 99°C in 0.5 s increments. All samples were run in triplicate, in parallel for each individual muscle sample and simultaneously with RNA-negative controls. Cyclophilin A (*Ppia*), glyceraldehyde 3-phosphate dehydrogenase (*Gapdh*) and Beta-actin (*Actb*) were tested as candidate reference genes being the latter the most stable to normalize Ct values by ΔCt method. Same data trends were obtained if *Ppia* or *Gapdh* were used (data not shown).

### Statistics

Data are expressed as means ± SE. Statistical differences between groups were determined by unpaired t-test (GraphPad software). Differences were considered statistically significant at the p < 0.05 level of confidence.

## Results

### Morphological analysis revealed microgravity-induced muscle atrophy mainly in *soleus*

**Fig 1:**
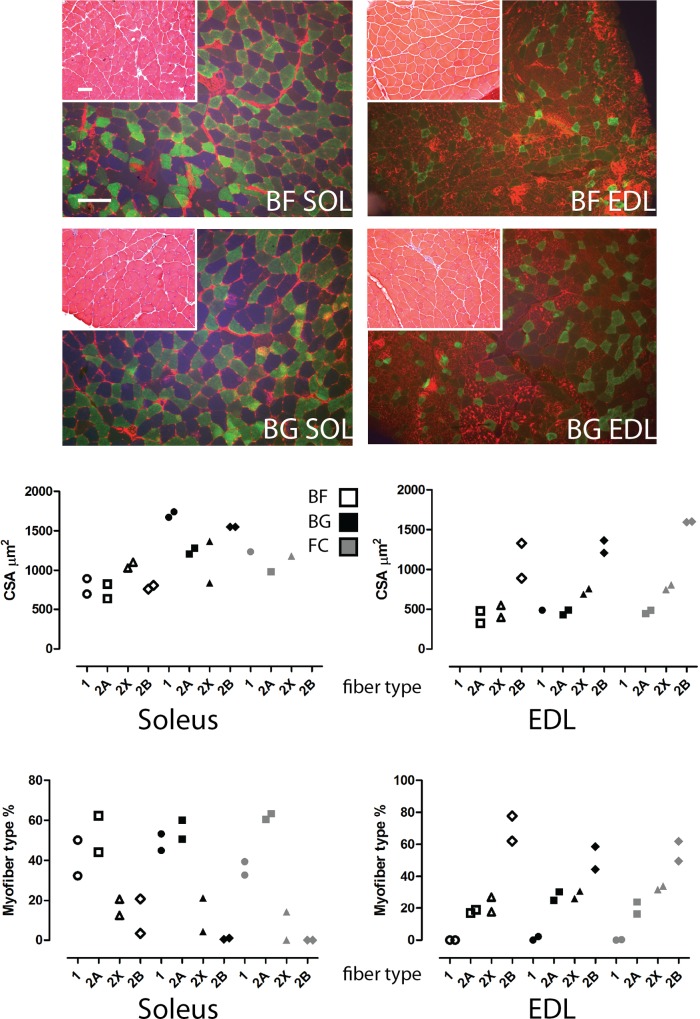
Morphological analysis of mouse *soleus* and EDL skeletal muscle flown on board the BION-M1 biosatellite for 30 days. Upper panel, insets show light microscopy images of Haematoxylin Eosin merged to immunofluorescence images of *soleus* (SOL) and EDL stained for MyHC isoforms (MyHC I: blue, IIa: green, IIb: red) in flown (BF) and control (BG) mice. Lower panel, scatter plots showing the quantification of the myofiber cross sectional area (CSA) and type composition in *soleus* (n = 2) and EDL (n = 2). Scale bar: 100 μm.

19–20 weeks old male C57BL/N6 mice were randomly divided in 3 groups: mice to be flown aboard the BION-M1 capsule exposed for 30 days to microgravity (BION Flown = BF), mice housed for 30 days under the same biosatellite bio-parameters (i.e., number of animals per group and identical housing conditions in a BION-M1 used habitat) on ground (BION Ground = BG), and mice housed in the animal facility concomitant to the duration of the biosatellite flight (FC = Flight Control). The histological analysis from hematoxylin-eosin stained cryosections showed the absence of major pathological features, such as central nuclei, immune cell infiltration or myofiber degeneration in all analyzed muscles from either of the experimental groups (BF, BG and FC) (Fig 1 and S1 Fig).

Immunofluorescence analysis was performed by means of antibodies recognizing dystrophin (a specific marker of the myofiber sarcoplasm membrane), and the main different myosin heavy chain isoforms (MyHC I, IIa, IIb subtypes found in murine muscle) to evaluate whether 30 days microgravity exposure affected the myofiber size (cross sectional area, CSA) and the myofiber phenotype composition in *soleus* and EDL (Fig 1 upper panel). Type IIx myofibers were identified by the absence of immunoreactivity in triple immunostained cryosections. As shown in Fig 1 (lower panel), the CSA of all myofiber types (I, IIa, IIx and IIb) was reduced in *soleus* of BF compared to BG, while the CSA of type I and IIa fibers was decreased in BF vs. FC. Conversely, in the EDL a reduction of the CSA was detected only in fibers expressing MyHC IIx of BF compared to BG mice, while the CSA of both MyHC IIx and IIb expressing myofibers was reduced in EDL of BF vs. FC mice.

Taken together, the analysis of MyHC isoform composition in flown vs. ground control muscles showed an increase in the percentage of MyHC IIb fibers in both *soleus* and EDL muscles of mice exposed to microgravity (BF) compared to ground control groups (both BG and FC), while a reduction in the percentage of MyHC I positive fibers was detected in the *soleus* of BF vs. BG mice only. Notably, no substantial additional changes in the proportional distribution of MyHC expressing fibers in EDL muscle were detectable in this study. As expected 30 days of microgravity exposure in the biosatellite particularly affected the *soleus* muscle of mice compared to the ground control animals.

### Transcriptome adaptation to microgravity exposure

In order to identify some of the molecular targets affected by microgravity exposure, we performed an expression profile analysis of *soleus* and EDL muscles from space flown vs. ground control mice. *Soleus* and EDL muscles of the three different experimental groups (BF, BG and FC) were analysed using Affymetrix mouse GeneChip array. In detail, we analysed 18 muscles, 9 solei and 9 EDL (BF n = 3, BG n = 3 and FC n = 3 for each muscle type). Gene expression profiles of muscle tissue deriving from all the different groups were compared to identify genes significantly differentially regulated in *soleus* and EDL (comparisons: BF vs. BG, FC vs. BG and BF vs. FC). More in detail, the comparison between BF vs. BG and BF vs. FC reflected the gene expression adaptation in skeletal muscle exposed to microgravity. In fact, the expression profile of BF mice was compared with the profiles of the two ground control groups (BG and FC). A comparison of the gene expression profiles of two ground control groups, FC vs. BG, was needed in order to rule out gene expression changes originating from different housing conditions (BG = replicate of the flight habitat and FC = animal facility habitat i.e. standard sized animal cages) of mice.

**Fig 2:**
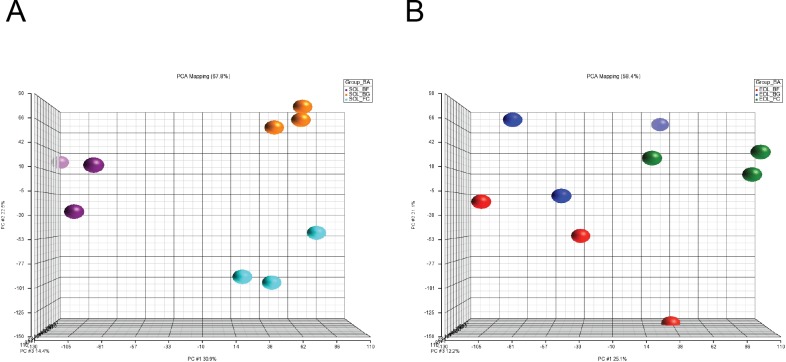
Principal Component Analysis (PCA) of gene expression data in *soleus* and EDL. PCA analysis of gene expression data in *soleus* (A) and EDL (B) highlights the high sensitivity of soleus to microgravity exposure compared to EDL.

Principal component analysis (PCA) was used to identify and compare the major effects of microgravity exposure on the gene expression regulation in *soleus* and EDL. In *soleus* all samples in the individual groups (BF, BG, FC) were found to be closer together, but all groups were found to be further apart and consequently showed larger dissimilarities across the genome (Fig 2A). In *soleus* the first axis (PCA1) explains about 1/3 (31%) of the difference in the data sets and is mostly related to changes of BF versus BG and FC. Dissimilarities between BG and FC in *soleus* are extracted by PCA2 and almost 1/4 of the variation in the entire data set. Thus the difference in BG versus FC is smaller than between BF versus BG and FC, respectively. In EDL, the PCA 1 axis represents only 25% of the variation. As seen in Fig 2B, the variances within all groups are more pronounced and differences between groups are smaller, thus they are not distinguishable between groups presented in PCA plot. These results confirmed greater effects of microgravity on *soleus* compared to EDL.

**Fig 3:**
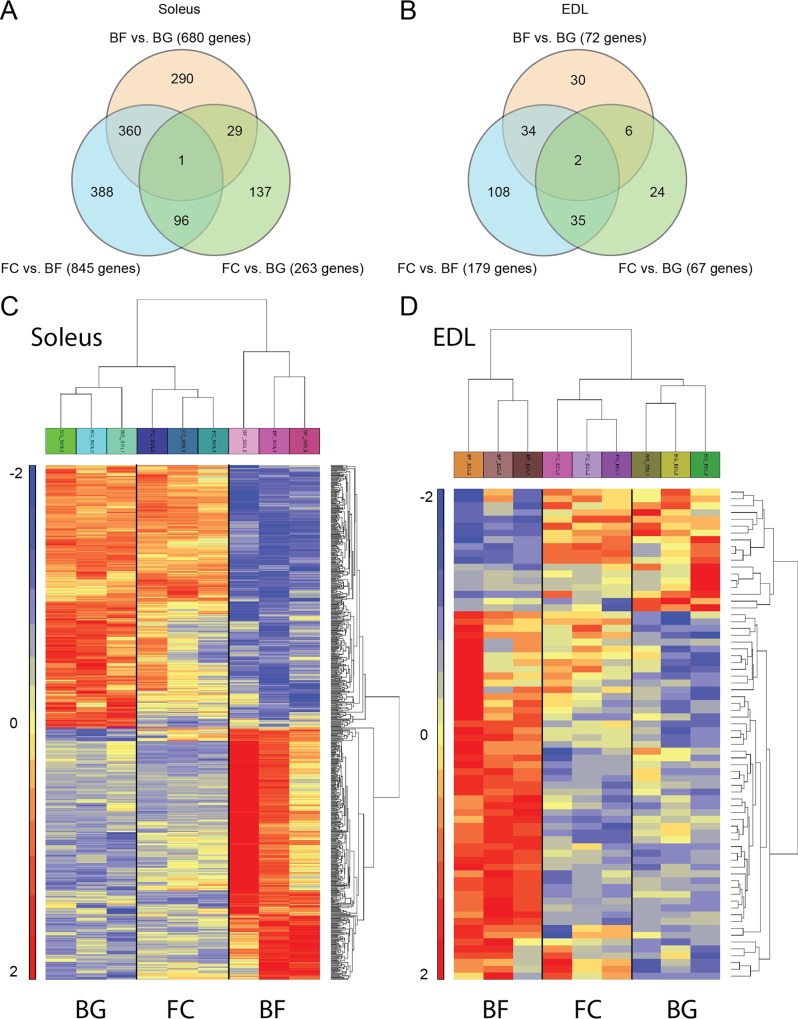
Venn diagrams and heat maps showing genes differentially regulated in *soleus* and EDL of BION-M1 space flown mice. A, B, Venn diagrams showing the number of genes differentially regulated comparing space flown (BF) with ground controls (BG and FC) in *soleus* (A) and EDL (B). Comparisons between the three different experimental groups are presented (BF vs. BG, FC vs. BG and BF vs. FC). C, D, hierarchical clustering centred on BF vs. BG in *soleus* (C, ordered BG-FC-BF) and EDL (D, ordered BF-FC-BG). The differentially regulated genes meeting FDR < 0,05 (*soleus*), p < 0.05 (EDL) and < -2 & > +2 fold change criteria are included in the heat maps.

Affymetrix data were filtered by means of the one way ANOVA statistical test (p < 0.05) and genes with fold change values greater than two (Fc +2) or smaller than minus two (Fc -2) were considered differentially expressed. The high number of differentially regulated genes in *soleus* of space flown mice (BF vs. BG) compared to EDL allowed us to apply a higher stringency to *soleus* analysis (False Discovery Rate < 0.05). The choice of applying two different stringencies to the analysis of *soleus* and EDL was rationale to select a suitable number of differentially regulated genes also for EDL muscle and to identify genes commonly regulated between the two different types of muscles.Thus, we considered as significantly differentially regulated those transcripts meeting the following cutoffs: **EDL**, fold change < -2 or > 2 unadjusted p value < 0.05; ***soleus***, fold change < -2 or > 2 FDR < 0.05. Venn diagrams in Fig 3A and 3B show the number of genes differentially and significantly regulated (meeting the above described cutoffs) in the three comparison (BF vs. BG, FC vs. BG and BF vs. FC) for *soleus* and EDL muscles. Comparing BF vs. BG, a total of 680 genes were differentially regulated in *soleus* muscle (334 up-regulated and 346 down-regulated), while only 72 genes were differentially regulated in EDL (54 up-regulated and 20 down-regulated). Comparing BF with FC (mice housed in standard mouse cages in the animal facility on the ground), 845 genes were differentially regulated in *soleus* (430 up-regulated and 415 down-regulated) while change in the expression of only 179 genes were detected in EDL (102 up-regulated and 77 down-regulated). Finally, comparing the two ground control groups (FC vs. BG), 263 genes resulted to be differentially regulated in *soleus* (85 up-regulated and 178 down-regulated), and 67 genes to be differentially regulated in EDL (41 up-regulated and 26 down-regulated).

The high number of genes differentially regulated in *soleus*, obtained by comparing the spaceflight group (BF) with both ground control groups (BG and FC), suggested that microgravity strongly affected muscular gene expression in the main postural muscle of lower limb (*soleus*) and only slightly changed muscular gene expression in EDL. On the other hand, the relatively low number of transcripts commonly regulated comparing BF vs. BG and FC vs. BG (interception between the two comparisons shown in the Venn diagrams, Fig 3A and 3B) in both *soleus* (30 genes) and EDL (8 genes) suggests that the different environments in which mice were housed in the two ground control groups (BG = replicate of the flight habitat and FC = animal facility habitat) only marginally affected gene expression changes found in the BF vs. BG comparison. Therefore, we next focused the subsequent analysis mainly to genes differentially regulated in the BF compared with BG (BF vs. BG).

Hierarchical clustering was used to group differentially regulated genes (Fc<-2/>2) in BG vs. BF of soleus (FDR < 0.05), and EDL (Fc<-2/>2; p < 0.05). Clustering was performed on 680 genes from SOL and 72 genes from EDL (rows) and three samples per BG, FC and BF, respectively (columns).

Two way hierarchical clustering analysis obtained comparing BF to BG showed similarity in the gene expression of the two ground controls (BG and FC) compared to microgravity exposed mice (BF) in both *soleus* and EDL. As shown in Fig 3C and 3D, BG and FC samples were arranged in the same cluster.

### Functional gene clusters and signaling pathways affected by spaceflight in mouse *soleus* and EDL muscles

To identify functional gene clusters specifically involved in microgravity-induced skeletal muscle adaptation, pathway enrichment and gene ontology (GO) categories were obtained by using GO enrichment tool of Partek^®^ Genomics Suite^®^ software and DAVID databases. Since the main aim of our study was to identify molecular players involved in the adaptation process induced by extended microgravity, we therefore centred our analysis on BF vs. BG comparison.

**Fig 4:**
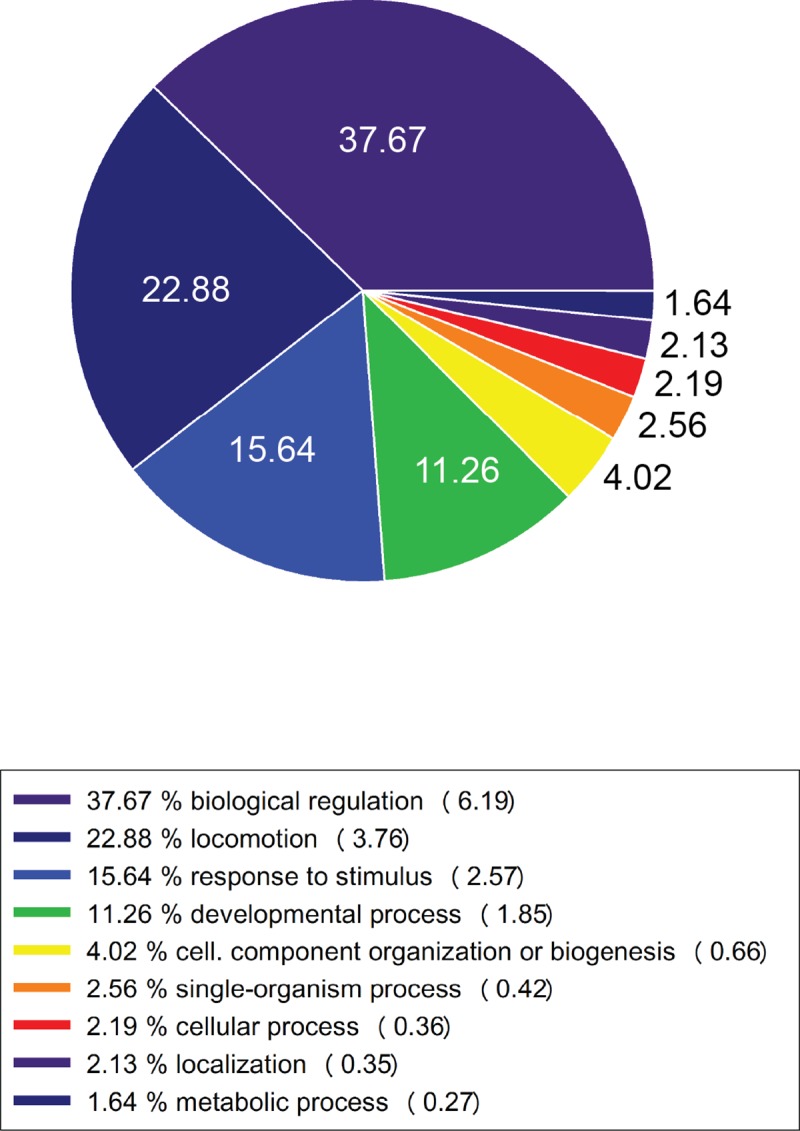
Biological functions regulated in *soleus* of BION-M1 flown mice. Pie graph showing the percentage of genes linked to biological functions differentially expressed in *soleus* in BF group compared to ground control (BG) group. GO enrichment tool of Partek^®^ Genomics Suite^®^ software was used to perform the analysis. The Fisher's Exact test on the counts of genes was used to identify key functional groups in biological functions with respect to an enrichment p value < 0.05 and more than 8 genes per group.

As shown in Fig 4, the GO analysis centred on 680 genes differentially regulated in *soleus* of mice exposed to microgravity (BF) compared to the ground control (BG) identified different GO biological key functional categories. Within the biological key functional categories identified, 37,67% of these genes resulted linked to the biological regulation processes, 22,88% to locomotion, 15,64% to response to stimulus, 4,02% to cellular component organization or biogenesis, 2,56% to single-organism processes, 2,19% to cellular processes, 2,13 to localization and 1,64% to metabolic processes.

Furthermore, DAVIDbased GO analysis identified 12 main functional gene clusters, plus a separate group of other genes of potential interest, affected by microgravity exposure in *soleus* (gene linked to functional clusters short listed in Table 1 and complete gene list in Supporting Information, S1, S2 and S3 Tables): contractile myofiber and regulation of muscle contraction, glucose and fatty acid metabolism, inflammatory response, calcium ion homeostasis, stress response, muscle development, response to oxidative stress, regulation of programmed cell death and focal adhesion. By contrast, only four functional gene clusters, plus a separate group of other genes of potential interest, were found in EDL: intermediate filaments, response to peptide hormone stimulus, acute inflammatory response and transcription factors activity (Table 2).

The major part of genes listed in the S1, S2 and S3 Tables were significantly and differentially regulated in the space-flown group (BF) compared to ground controls (BG and FC) in *soleus*. When the two ground controls were compared (FC vs. BG), the expression of about 85% of genes were not significantly changed, suggesting that the difference in mice housing conditions (standard cage vs. flight habitat) only marginally affected the transcriptional regulation outcome of genes induced by microgravity exposure. Among all the genes included in the complete list, only 13 genes (10%) were differentially regulated in EDL (BF vs. BG), thus showing a high muscle-specific transcriptome adaptation, very likely induced by microgravity in spaceflight. On the other hand, gene enrichment analysis in EDL (Table 2) showed a more heterogeneous outcome in which the expression of 40% of the genes (11 of 27 genes) significantly regulated in BF vs. BG were, however, not significantly changed in BF vs. FC. This would indicate that the observed changes in EDL gene expression could be attributed to the two different on the ground mice housing conditions (i.e., standard cage vs. replica of flight habitat) between BG and FC. Nevertheless, only 2 of them were significantly differentially regulated comparing BG with FC.

Moreover, pathway analysis using KEGG module of WEB-based GEne SeT AnaLysis Toolkit [26] focusing only on the genes significantly differentially regulated in BF vs. BG of *soleus* and EDL was performed to assess which signalling pathways were mainly affected by 30 days of microgravity (Table 3).

**Table 3:** Signaling pathways differentially regulated in *soleus* and EDL of BION-M1 space flown mice.

	Pathway Name	# Gene	Entrez Gene	Statistics
*soleus*	PPAR signaling pathway	13	104086 20411 22190 16956 11430 113868 12491 11450 19013 1183214077 14081 12896	R = 4.47;rawP = 4.94e-06;adjP = 0.0006
	Peroxisome	12	18631 28200 11430 113868 269951 26874 14081 70503 17117 1235915488 13850	R = 3.95;rawP = 4.21e-05;adjP = 0.0027
	Fatty acid metabolism	8	11430 113868 110446 231086 14081 52538 97212 12896	R = 4.85;rawP = 0.0002;adjP = 0.0065
	Adipocytokine signaling pathway	10	72674 19017 12491 11450 19013 108099 19082 14081 20528 16846	R = 3.84;rawP = 0.0002;adjP = 0.0065
	Nitrogen metabolism	5	27053 14645 23831 107869 12319	R = 5.76;rawP = 0.0014;adjP = 0.0301
	Propanoate metabolism	6	16832 97212 20917 227095 110446 60525	R = 4.77;rawP = 0.0013;adjP = 0.0301
	Protein digestion and absorption	9	11931 12814 12825 98660 11932 12842 20514 12830 11928	R = 3.00;rawP = 0.0028;adjP = 0.0413
	MAPK signaling pathway	20	13537 14164 12299 19043 17869 18479 18750 15507 17762 63953 1804968794 225028 66350 19042 110651 14281 66922 26410 15481	R = 1.94;rawP = 0.0036;adjP = 0.0413
	Proximal tubule bicarbonate reclamation	4	11931 98660 11932 11928	R = 5.76;rawP = 0.0042;adjP = 0.0413
	ECM-receptor interaction	9	12814 12845 12491 12825 12505 16782 12842 12830 12643	R = 2.80;rawP = 0.0046;adjP = 0.0413
EDL	ErbB signaling pathway	3	12575 13685 18708	R = 9.02;rawP = 0.0045;adjP = 0.0473
	PPAR signaling pathway	3	20249 20411 11450	R = 9.96;rawP = 0.0034;adjP = 0.0473
	mTOR signaling pathway	2	13685 18708	R = 10.34;rawP = 0.0159;adjP = 0.0581
	Type II diabetes mellitus	2	11450 18708	R = 10.11;rawP = 0.0166;adjP = 0.0581
	Insulin signaling pathway	3	20411 13685 18708	R = 5.90;rawP = 0.0142;adjP = 0.0581
	Jak-STAT signaling pathway	3	16194 12804 18708	R = 5.61;rawP = 0.0163;adjP = 0.0581
	Glioma	2	12575 18708	R = 8.39;rawP = 0.0236;adjP = 0.0604
	Phosphatidylinositol signaling system	2	20975 18708	R = 7.80;rawP = 0.0271;adjP = 0.0604
	Melanoma	2	12575 18708	R = 6.95;rawP = 0.0335;adjP = 0.0604
	Chronic myeloid leukemia	2	12575 18708	R = 6.84;rawP = 0.0345;adjP = 0.0604

Differentially regulated genes (BF vs. BG) in *soleus* (FDR<0.05 and < -2 & > 2 fold change) and EDL (p value < 0.05 (2 way ANOVA) and < -2 & > 2 fold change) were analysed by KEGG module of WEB-based GEne SeT AnaLysis Toolkit. The top 10 most significantly enriched pathways in each muscle were included in the table. RawP: p value from hypergeometric test and adjP: p value adjusted by the multiple test adjustment.

### Gene expression changes induced by microgravity exposure in both *soleus* and EDL

**Fig 5:**
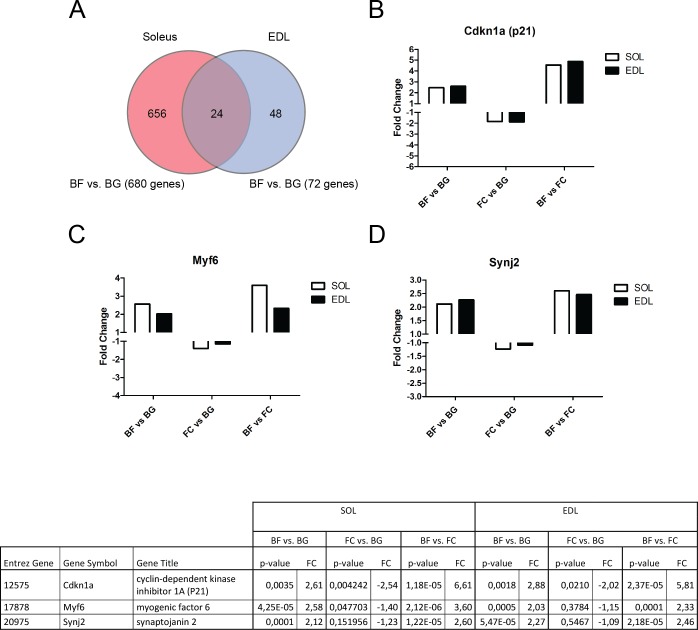
Common genes differentially regulated in *soleus* and EDL in BION-M1 flown mice. A, Venn diagram showing the number of genes differentially regulated in *soleus* and EDL following 30 days of microgravity exposure (BF vs. BG). B, C, D, bar charts showing the fold change in the expression of cyclin-dependent kinase inhibitor 1A (P21) (B), the myogenic factor 6 (C) and synaptojanin 2 (D) in both *soleus* and EDL muscles of flown mice (BF) compared to ground controls (BG and FC). The table includes the fold change and p-values for each gene depicted as bar graph in B-D.

In order to identify common genes regulated by microgravity in both *soleus* and EDL, genes differentially regulated between BF and BG were further compared in the two muscles. Among the 680 genes differentially regulated in *soleus* and the 72 genes regulated in EDL, the expression of only 24 genes were significantly changed in both muscles (Table 4) as shown in the Venn diagram in Fig 5A. The low number of commonly regulated genes in both *soleus* and EDL support the hypothesis that changes in gene expression in response to microgravity exposure are highly muscle-specific. Among the identified transcripts, two genes were linked to cell proliferation (cyclin-dependent kinase inhibitor 1A (P21) (Fig 5B) and CDK5 regulatory subunit associated protein 1-like 1), the myogenic factor 6 (Myf6) (Fig 5C) and synaptojanin 2 (Synj2) (Fig 5D) were strongly up-regulated, while v-maf musculo-aponeurotic fibrosarcoma oncogene family, protein B (Mafb) and uracil DNA glycosylase Ung resulted in strong down-regulation in the space flown group (BF) compared to both ground control groups (BG and FC).

**Table 4:** List of genes differentially regulated in both *soleus* and EDL in BION-M1 space flown mice.

				SOL						EDL			
		BF vs. BG		FC vs. BG		BF vs. FC		BF vs. BG		FC vs. BG		BF vs. FC	
Entrez Gene	Gene Symbol	p-value	*FC*	p-value	*FC*	p-value	*FC*	p-value	*FC*	p-value	*FC*	p-value	*FC*
18405	**Orm1**	7,98E-05	**5,59**	0,649953	**1,15**	0,0001673	**4,88**	0,019054	**2,22**	0,334438	**1,35**	0,11458	**1,65**
17918	**Myo5a**	1,10E-06	**5,43**	0,126039	**1,36**	8,75E-06	**3,99**	6,01E-07	**6,00**	0,286964	**1,23**	2,23E-06	**4,87**
15439	**Hp**	0,000688	**5,32**	0,148717	**1,77**	0,0113157	**3,01**	0,022807	**2,62**	0,022247	**2,63**	0,98947	**-1,00**
11450	**Adipoq**	0,000509	**5,05**	0,0537364	**2,09**	0,024636	**2,42**	0,002715	**3,65**	0,000696	**4,74**	0,45988	**-1,30**
16819	**Lcn2**	0,00222	**4,21**	0,436003	**-1,35**	0,0005342	**5,68**	0,000137	**7,70**	0,830056	**-1,08**	9,68E-05	**8,35**
72655	**Snhg5**	2,48E-08	**3,43**	0,169217	**-1,15**	7,25E-09	**3,95**	5,95E-06	**2,10**	0,490698	**-1,07**	2,40E-06	**2,25**
11433	**Acp5**	0,006445	**3,39**	0,359508	**1,42**	0,0374911	**2,38**	0,019126	**2,73**	0,333955	**1,45**	0,11517	**1,88**
83673	**Snhg1**	2,86E-07	**3,39**	0,000586	**-1,74**	4,42E-09	**5,88**	2,30E-05	**2,22**	0,160745	**-1,20**	3,06E-06	**2,65**
68916	**Cdkal1**	0,000924	**3,15**	0,92231	**1,03**	0,0011012	**3,07**	0,000994	**3,11**	0,625883	**1,14**	0,00244	**2,73**
11537	**Cfd**	0,003979	**3,13**	0,0412539	**2,08**	0,229282	**1,50**	0,017779	**2,42**	0,002263	**3,46**	0,28601	**-1,43**
12575	**Cdkn1a**	0,003504	**2,61**	0,0042418	**-2,54**	1,18E-05	**6,61**	0,001784	**2,88**	0,02099	**-2,02**	2,37E-05	**5,81**
17878	**Myf6**	4,25E-05	**2,58**	0,0477034	**-1,40**	2,12E-06	**3,60**	0,000532	**2,03**	0,378428	**-1,15**	0,00012	**2,33**
13685	**Eif4ebp1**	1,87E-06	**2,47**	0,981147	**1,00**	1,93E-06	**2,47**	6,84E-06	**2,22**	0,063676	**1,24**	0,00014	**1,79**
20975	**Synj2**	0,000119	**2,12**	0,151956	**-1,23**	1,22E-05	**2,60**	5,47E-05	**2,27**	0,546731	**-1,09**	2,18E-05	**2,46**
140742	**Sesn1**	7,73E-06	**2,11**	0,299864	**-1,11**	1,93E-06	**2,35**	3,53E-07	**2,72**	0,045714	**-1,25**	3,87E-08	**3,40**
18948	**Pnmt**	0,004559	**2,09**	0,732886	**-1,08**	0,002405	**2,25**	6,55E-05	**3,53**	0,471281	**-1,17**	2,16E-05	**4,14**
83673	**Snhg1**	0,001193	**2,08**	0,0698669	**-1,41**	4,53E-05	**2,95**	0,61843	**1,09**	0,428498	**-1,15**	0,20801	**1,26**
18162	**Npr3**	0,004588	**-2,16**	0,0082477	**-2,01**	0,756813	**-1,07**	0,014382	**1,89**	0,471607	**1,18**	0,05597	**1,60**
20411	**Sorbs1**	0,008884	**-2,44**	0,265102	**-1,40**	0,0750276	**-1,75**	0,029162	**2,03**	0,096792	**1,67**	0,51265	**1,21**
66695	**Aspn**	0,000752	**-2,52**	0,00928	**-1,89**	0,191014	**-1,33**	0,002575	**2,19**	0,865739	**-1,04**	0,00188	**2,27**
22256	**Ung**	5,71E-06	**-3,15**	0,511389	**1,11**	2,40E-06	**-3,49**	0,000365	**-2,08**	0,26731	**1,19**	5,64E-05	**-2,48**
16658	**Mafb**	0,001353	**-3,47**	0,99476	**-1,00**	0,0013688	**-3,47**	0,000479	**-4,15**	0,646026	**-1,15**	0,00109	**-3,60**
22042	**Tfrc**	6,58E-05	**-3,62**	0,140757	**-1,41**	0,0008869	**-2,58**	0,005811	**-2,06**	0,613272	**-1,12**	0,01523	**-1,84**
13170	**Dbp**	0,007662	**-3,84**	0,0096373	**3,65**	4,11E-05	**-14,01**	0,029217	**-2,83**	0,066869	**2,33**	0,00074	**-6,62**

The genes differentially regulated (BF vs. BG) in both *soleus* and EDL meeting < -2 & > 2 fold change criteria were included in the table.

### Validation of selected differentially regulated genes in *soleus* of mice exposed to microgravity

**Fig 6:**
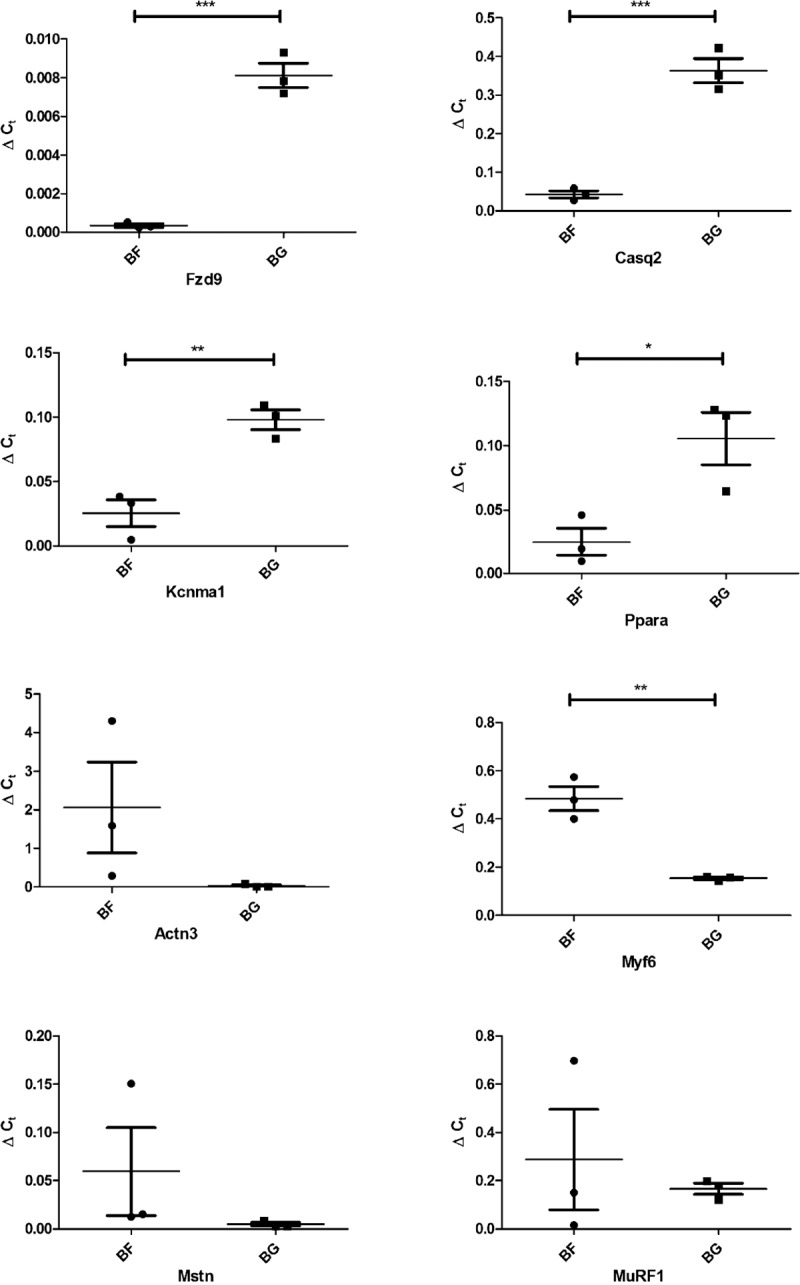
Real time qPCR analysis of selected genes differentially regulated in *soleus* of BF vs. BG mice. Expression levels of frizzled homolog 9 (Fzd9), calsequestrin 2 (Casq2), potassium large conductance calcium-activated channel, subfamily M, alpha member 1 (Kcnma1), peroxisome proliferator activated receptor alpha (Ppara), actinin alpha 3 (Actn3), myogenic factor 6 (Myf6), myostatin (Mstn) and Muscle RING Finger 1 (MuRF1) were evaluated by real-time quantitative PCR in soleus of flown (BF) and ground control mice (BG). *Actb* (beta actin) was used as housekeeping to calculate the delta Ct of the selected genes. Graph shows ΔC_t_ ± SEM; *** p < 0,0007, ** p < 0,006 and * p < 0,03.

To validate gene expression changes observed following 30 days of microgravity by means of Affymetrix analysis, quantitative real time PCR (qPCR) was performed. Given that *soleus* muscle is highly affected by microgravity exposure compared to EDL, we focused the validation only on this muscle. The choice of genes to be validated was based on the low p values, fold changes > 2.5 and potential involvement in skeletal muscle physio-pathology. Due to the low amount of tissue used for RNA extraction, only the expression of 6 genes were evaluated by qPCR in flown mice (BF) compared to ground control (BG): frizzled homolog 9 (*Fzd9*), calsequestrin 2 (*Casq2*), potassium large conductance calcium-activated channel, subfamily M, alpha member 1 (*Kcnma1*), peroxisome proliferator activated receptor alpha (*Ppara*), actinin alpha 3 (*Actn3*) and myogenic factor 6 (*Myf6*). S4 Table includes the sequence of the primer used in qPCR analysis. Three housekeeping genes were used as reference to calculate the delta Ct of the selected genes: *Actb*, beta actin; *Ppia*, cyclophilin A; *Gapdh*, glyceraldehyde-3-phosphate dehydrogenase. As shown in Fig 6, qPCR data normalized using Actb showed that Fzd9, Casq2, Kcnma1, Ppara and Myf6 were significantly differentially regulated in *soleus* of BF compared to BG mice, confirming the reliability of the Affymetrix analysis for the selected genes. More in detail, 30 days microgravity exposure induced a significant reduction in Fzd9, Casq2, Kcnma1 and Ppara expression, and a significant increase in Myf6 expression in *soleus* of flown mice. Similar results were obtained using GAPDH (data not shown) and Ppia (S2 Fig) as housekeeping genes.

Myostatin (Mstn) and Muscle RING Finger 1 (MuRF1) are both well-known regulators of skeletal muscle mass, but unfortunately the probes targeting these transcripts were not included in the Affymetrix Mouse Genome 430A 2.0 Array. Thus, microgravity-induced gene expression changes of myostatin (Mstn) and MuRF1were evaluated by qPCR (Fig 6 and S2 Fig). Mstn transcript tended to increase in soleus of flown mice (BF) compared to BG mice, while mRNA level of MuRF1 was not changed. Nevertheless, both these transcripts turned out not to be significantly regulated in the *soleus* of space-flown mice compared to ground controls.

## Discussion

The transcriptome profile established by the present study provides a new and comprehensive yet missing overview on the microgravity effects on the global gene expression in the slow-type *soleus* vs. the fast type EDL of space-flown mice. To our knowledge, this is the first study in which *soleus* and EDL collected from long-duration 30 days space-flown mice were analysed using microarray technology. Because of its mainly slow phenotype (rat and mice) and due to its known susceptibility to unloading and disuse, the antigravity and postural calf *soleus* has often been studied in experimental models of unloading to investigate signaling pathways involvement in skeletal muscle atrophy [28, 29], aging mechanisms of sarcopenia [30, 31], microgravity effects on skeletal muscle [3, 7, 32], and skeletal muscle structural and functional “integrity” in bed rest studies [33]. Considering the very high susceptibility of the *soleus* to disuse on Earth and to extended weightlessness in space compared with other fast-phenotype hind limb muscles reported from previous rodent spaceflight experiments (e.g., *gastrocnemius*, *anterior tibialis*, *plantaris*), further knowledge on the complete gene expression profile of this particular antigravity calf muscle obtained from space-flown mice are key to a deeper insight of the molecular mechanism of microgravity-induced skeletal muscle atrophy. Moreover, the comparison of the individual gene array data between *soleus* and EDL muscles showed that long duration microgravity exposure mainly affected global gene expression in *soleus*, whereas in EDL a much smaller number of genes was differentially expressed after spaceflight. This finding highlights the muscle-specificity of the microgravity-induced transcriptome rearrangement in these two very different muscle types from the lower limbs of space-flown mice.

Another important asset of the present experimental design is the comparison of the flown mice (BF) with two different age- and sex-matched ground controls (BG and FC) to minimize any bias related to housing conditions and animal treatment.

### Structural microgravity effects on space-flown skeletal muscle

Previously published results on the 2013 BION-M1 mission reported some structural protein changes (alpha-actinin-1, beta-actin) in space-flown mice *soleus* and *tibialis anterior* [18], the slow to fast fiber phenotype shift and decrement of titin and nebulin functional proteins in the space-flown *gastrocnemius* and *tibialis anterior* [19]. In our study we also found reduced CSAs observed in type I, IIa, IIb and IIx myofibers in *soleus* of mice flown aboard of the biosatellite BION-M1 compared with ground based controls, and thus largely confirmed the notion that the postural muscles of lower limbs were highly responsive to microgravity unloading [3, 34, 35]. We also performed immunohistochemistry analysis with a set of MyHC slow and fast subtype-specific antibodies that showed an expected myofiber type shift from slow to fast mainly in *soleus* muscle flown in space for 30 days on board the biosatellite. These results were further supported by transcriptome analysis, showing a robust upregulation of fast specific genes such as *Myh4*, coding for the fast MyHC IIb, and the fast actinin alpha 3 in *soleus* muscle of mice exposed to microgravity.

Our morphological data support earlier findings of a reduction of the CSA in all fiber types in *soleus*, *gastrocnemius* and *plantaris*, and an increase of the fast type fiber composition exclusively in *soleus* of mice exposed to microgravity for 11 days on a NASA Shuttle mission [5]. In general, the 30 days BION-M1 space-flown muscles investigated in the present study already showed comparable structural changes (reduced CSA and fiber type shift) that have been reported in normal mouse *soleus* flown for as long as 91 days (MDS mission, 2009) on board the International Space Station [7].

### Microgravity effects on muscular gene expression: ground vs. spaceflight effects

In the 30 days BION-M1 space-flown *soleus* we found as much as 680 significantly differentially regulated genes (comparing BF vs. BG) while only 72 genes were found in EDL, including some yet unknown microgravity sensitive gene transcripts. Most of the newly identified transcripts are linked to various key biological functions and mechanisms (contractile machinery, metabolism, inflammatory and stress responses). Muscle specific changes in gene expression were previously reported mainly from the mouse and rat fast-type *gastrocnemius* following 11, 16 or 17 days of short-duration spaceflight [14–16]. Allen and co-workers found a significant down-regulation of peroxisome proliferator-associated receptor (PPAR) γ coactivator-1α (PPARGC-1α, PGC-1α) and peroxisome proliferator activated receptor alpha (PPARα) in 11 days space-flown mice gastrocnemius onboard of the STS108 shuttle [14]. However, we found a more complex pattern of peroxisome proliferator-associated receptor (PPAR) γ coactivator-1β (PPARGC-1β, PGC-1β) and peroxisome proliferator activated receptor alpha (PPARα) that both were strongly down-regulated in the space-flown *soleus*, but not in the EDL, compared to ground controls. PGC-1 coactivators are involved in skeletal muscle fiber type specification and that overexpression of these genes increased the myofiber oxidative metabolism [36]. More recently, other laboratories demonstrated that the PGC-1 coactivators deficiency is crucial for exercise performance, mitochondrial structure and function, but surprisingly not for myofiber specification [37].

Beyond the canonical role of PGC-1 in normal muscle plasticity and adaptation, it has been recently demonstrated that these coactivators are both able to reduce the pro-inflammatory cytokines production induced by TNFα or TLR agonists in normal skeletal muscle on the ground [38, 39]. We thus observed an increase in the expression of transcripts of several inflammatory chemokines in the *soleus* of the space-flown mice suggesting that components of the inflammatory response may play a role during extended microgravity-induced muscle atrophy. For example, we observed a strong up-regulation of gene transcripts coding for chemokines such as Ccl2, Ccl7 and Ccl12, that are ligands of the CC Chemokine receptor 2 (CCR2), crucially involved in the recruitment of monocytes/macrophages at the inflammatory sites [40]. Notably, we did not find immune cell infiltrates in the flown skeletal muscle, suggesting that the time frame under investigation, i.e. 30 days of microgravity, was probably only the beginning of a more complex process involving the cellular immune response in ongoing muscle atrophy in microgravity. We thus may only speculate that alternative components of the inflammatory machinery might be, at least in part, responsible for the observed microgravity-induced muscle atrophy/wasting observed in BION-M1 experiment. Recently, an increase of the concentration of CCL2 and other inflammatory cytokines was actually found in the blood plasma of astronauts following long term space flight [41], but a rationale for this observation remains to be determined yet. Further studies are required in space research to unequivocally address the still open question of an inflammatory response hypothesis put forward in this work.

In our study, we observed that Casq2 transcript was strongly downregulated in mice exposed to microgravity. Calcium signaling is without doubt crucial for the physiology of excitation-contraction coupling in skeletal muscle [42]. Intracellular Ca^2+^ is stored in the sarcoplasmic reticulum (SR), and in skeletal muscle it is released mainly by the terminal cisternae (TC). Calsequestrin (Casq) is the main Ca^2+^ binding protein located in the SR lumen; it has a pivotal role in both in Ca^2+^ storage and in modulating Ca^2+^ release from ER [43]. It is known that two Casq isoforms are expressed in skeletal muscle: Casq1, the skeletal muscle specific isoform, and Casq2, the cardiac isoform. Casq1 and 2 are both expressed in slow skeletal muscle fibers, whereas only Casq1 is expressed in fast fibers [44]. Since Casq2 is strictly expressed in the slow muscle fibers, our result likely reflects the slow to fast phenotype switch (reduced Casq2 transcripts) observed in fibers of the space-flown mice *soleus*.

We also observed that Fzd-9 expression was strongly downregulated in space-flown soleus compared to ground controls. Wnts ligands and their respective receptors, members of the Frizzled family, are responsible for essential developmental and homeostasis processes through multiple pathways in laboratory experiments[45]. Recently, Frizzled 9 homolog (Fzd9) has been localized specifically at the postsynaptic region of neuro-muscular junctions in mouse skeletal muscle possibly involved in the clustering of acetylcholine receptor [46]. Our results agree with previous studies showing decreased expression of Fzd9 gene in *gastrocnemius* muscle after denervation or spaceflight [14, 47] and thus confirmed a potential efficacy of Fzd9 as a marker of microgravity induced skeletal muscle adaptation also in the space-flown *soleus* muscle.

Myogenic regulatory factors (MRFs) have a pivotal role in the development and differentiation of skeletal muscle. Among MRFs, MyoD and Myf5 have a crucial role in skeletal myogenic specification, while myogenin and MRF4 (Myf6) are involved in differentiation as demonstrated by their temporal pattern of expression in embryos [48, 49]. The present microarray data from the BION-M1 experiment showed that MyoD, myogenin and MRF4 were upregulated in *soleus* of space-flown mice, whereas only MRF4, not MyoD was upregulated in the EDL following 30 days of microgravity. We selected only MRF4 for real time PCR validation, confirming the observed change in mRNA expression of this gene in the *soleus* of flown mice. Although the role of MRF4 in adult skeletal muscle is still elusive, it is known that denervation and devascularization induce a nuclear localization of MRF4 in adult myofibers independently from the type I or II phenotype, suggesting that MRF4 is involved in the gene expression reprogramming in denervated and regenerating muscle [50, 51]. In a recent laboratory experiment, MRF4 knockdown in rodent *soleus in vivo* induced hypertrophy and prevented denervation-induced atrophy in adult skeletal muscle by controlling MEF2 activity [52]. Our present results suggest that MRF4 and other MRFs also may play a crucial role in the myofiber response to microgravity-induced atrophy.

We also observed a downregulation of Kcnma1 expression in the space-flown *soleus* of mice. Potassium calcium-activated channel subfamily M alpha 1 (Kcnma1), also called large-conductance Ca^2+^ -activated K^+^ (BK_Ca_) channel, is a K^+^ channel that can be activated alone or synergistically by both membrane depolarization or intracellular Ca^2+^ [53]. In skeletal muscle, KCNMA1 is known to be involved in the modulation of excitability [54]. Moreover, different splicing variants of BK_Ca_ channel are specifically expressed in different muscles, consequentially conferring distinctive biophysical and pharmacological proprieties [55] which in turn can be modulated by hind limb unloading [54] and could be also regulated by microgravity.

Myostatin (Mstn) is known to regulate muscle mass in mice [56], but unfortunately the probes targeting these transcripts were not included in the hybridized microarray. Thus, we evaluated potential Mstn gene expression changes by real time qPCR, showing that in *soleus* this transcript inclined to increase in space-flown mice compared to ground controls. Our results match with previous results, showing a non-significant increase in the mRNA level of Mstn in the *gastrocnemius* exposed to microgravity [14]. On the other hand, qPCR analysis showed that in our study the levels of Muscle RING Finger 1 (MuRF1) mRNA, an ubiquitin ligase involved in the regulation of muscle mass [57], were not significantly changed in *soleus* of 30 days´ space-flown mice. Notably others have shown an increase of MuRF in *gastrocnemius* of rats following 16 days of microgravity [15], suggesting the presence of a variable and time-dependent pattern of MuRF expression level in microgravity that needs further investigation.

### Conclusion

The present study for the first time provides a novel and comprehensive overview on global gene expression adaptation to 30 days of microgravity exposure in mouse *soleus* and EDL muscles. The present dataset highlights a number of newly identified microgravity susceptible gene transcripts in the space-flown mice *soleus* linked to key biological processes crucially involved in normal skeletal muscle physiology. However further studies are needed to investigate the functional role of microgravity sensitive genes (gene by gene) found in spaceflight, for example by establishing transgenic or knockout mice models. Nevertheless, the systematic analysis of microgravity-sensitive muscle genes in the space-flown *soleus* now provides potential new targets or biomarkers for the development of new efficient countermeasures able to prevent, or at least in part minimize, microgravity-induced skeletal muscle atrophy with optimized physical exercise prescriptions in future crewmembers of spaceflights, that may be also applicable in various clinical settings and rehabilitation.

## Supporting Information

S1 Fig Haematoxylin Eosin staining of space flown mice and ground controls. Haematoxylin Eosin of *soleus* (SOL) and EDL in flown (BF) and control (BG) mice. Scale bar: 100 μm.
(TIF)

S2 Fig Real time qPCR analysis of selected genes differentially regulated in *soleus* of BF vs. BG mice. Expression levels of frizzled homolog 9 (Fzd9), calsequestrin 2 (Casq2), potassium large conductance calcium-activated channel, subfamily M, alpha member 1 (Kcnma1), peroxisome proliferator activated receptor alpha (Ppara), actinin alpha 3 (Actn3), myogenic factor 6 (Myf6), myostatin (Mstn) and Muscle RING Finger 1 (MuRF1) were evaluated by real-time quantitative PCR in soleus of flown (BF) and ground control mice (BG). *Ppia* (cyclophilin A) was used as reference to calculate the delta Ct of the selected genes. Graph shows ΔC_t_ ± SEM; ** p < 0,0075 and * p < 0,025.
(TIF)

S1 Table Functional gene clusters differentially regulated in soleus following 30 days of microgravity exposure (part 1). The differentially regulated genes (BF vs. BG) in soleus meeting FDR < 0.05 and < -2 & > 2 fold change criteria were analysed by DAVID database and the complete list of genes (part 1) linked to the main functional clusters is included in this table.
(PDF)

S2 Table Functional gene clusters differentially regulated in soleus following 30 days of microgravity exposure (part 2). The differentially regulated genes (BF vs. BG) in soleus meeting FDR < 0.05 and < -2 & > 2 fold change criteria were analysed by DAVID database and the complete list of genes (part 2) linked to the main functional clusters is included in this table.
(PDF)

S3 Table Functional gene clusters differentially regulated in soleus following 30 days of microgravity exposure (part 3). The differentially regulated genes (BF vs. BG) in soleus meeting FDR < 0.05 and < -2 & > 2 fold change criteria were analysed by DAVID database and the complete list of genes (part 3) linked to the main functional clusters is included in this table.
(PDF)

S4 Table Quantitative PCR primers and conditions. HK, Reference genes; bp, expected product size; T°, annealing temperature. * Sandonà D. et al. (2012) Adaptation of Mouse Skeletal Muscle to Long-Term Microgravity in the MDS Mission. PLoS ONE 7(3): e33232.
(PDF)

## Abbreviations

BF: BION flown
BG: BION ground
FC: flight control
EDL: extensor digitorum longus
CSA: cross sectional area
MyHC: myosin heavy chain
HE: haematoxylin-eosin
PCA: principal component analysis
FDR: False Discovery Rate
PPARGC-1α, PGC-1α: peroxisome proliferator-associated receptor (PPAR) γ coactivator-1α
PPARGC-1β,PGC-1β: peroxisome proliferator-associated receptor (PPAR) γ coactivator-1β
PPAR-α: peroxisome proliferator activated receptor alpha
Myh4: Myosin heavy chain 4
Casq2: calsequestrin 2
Myog: myogenin
Nqo1: NAD(P)H dehydrogenase quinone 1
Ccl2, Ccl12, Ccl7: Chemokine (C-C motif) ligand 2, 12, 7
CCR2: CC Chemokine receptor 2
Cat: catalase
TNFα: tumor necrosis factor alpha
TLR: Toll-like Receptor
FZD9: frizzled homolog 9
KCNMA1: potassium large conductance calcium-activated channel, subfamily M, alpha member 1
ACTN3: actinin alpha 3
MYF6: myogenic factor 6
SE: standard error
SEM: standard error of mean
