# Supplementary figures and images for: Gene Expression Profiling in Slow-Type Calf Soleus Muscle of 30 Days Space-Flown Mice
Source: PLoS One. 2017 Jan 11;12(1):e0169314. doi: 10.1371/journal.pone.0169314 (PMC5226721; doi:10.1371/journal.pone.0169314)

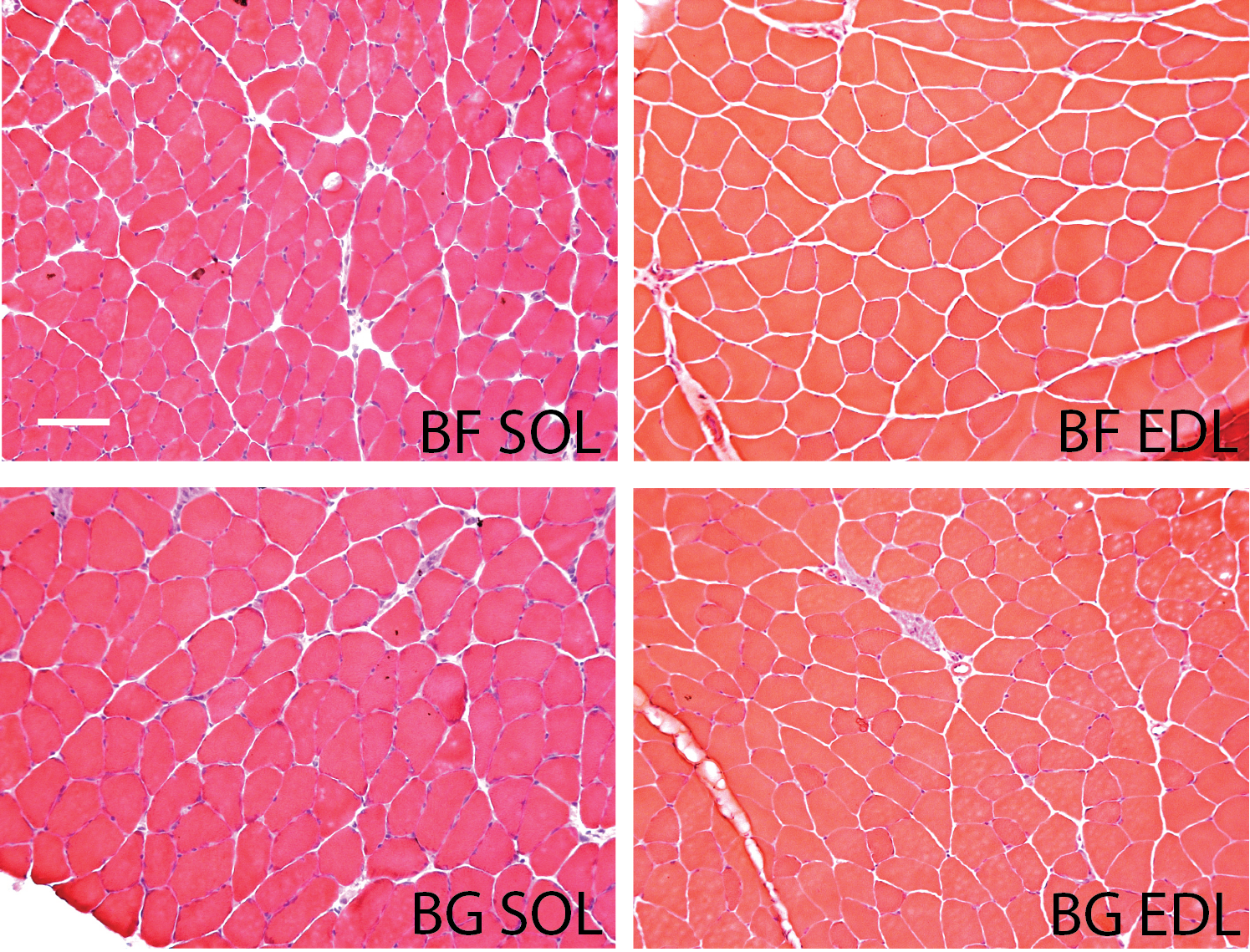

Supplement: S1 Fig — Haematoxylin Eosin of soleus (SOL) and EDL in flown (BF) and control (BG) mice. Scale bar: 100 μm. (TIF) [file pone.0169314.s001.tif]

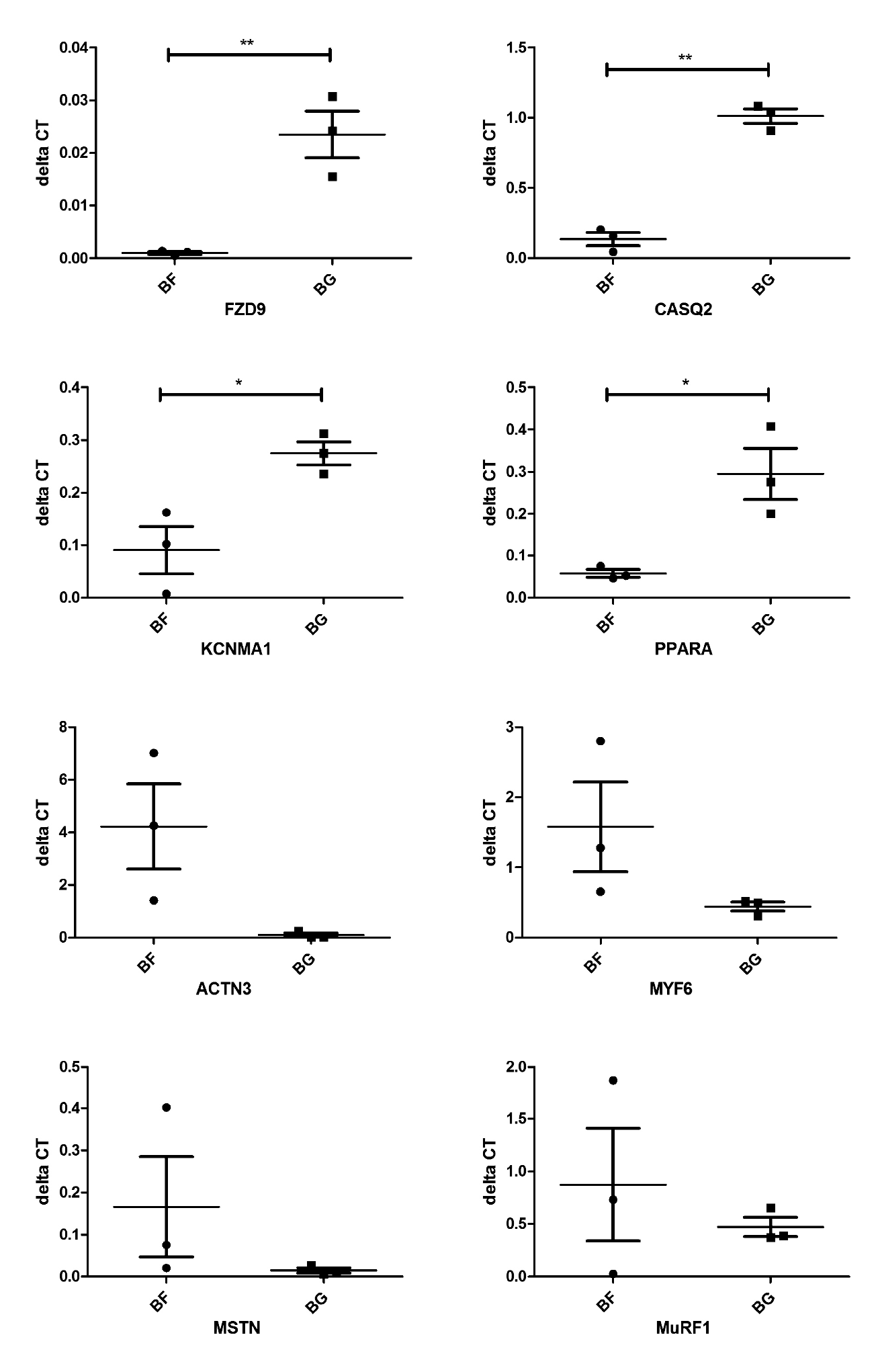

Supplement: S2 Fig — Expression levels of frizzled homolog 9 (Fzd9), calsequestrin 2 (Casq2), potassium large conductance calcium-activated channel, subfamily M, alpha member 1 (Kcnma1), peroxisome proliferator activated receptor alpha (Ppara), actinin alpha 3 (Actn3), myogenic factor 6 (Myf6), myostatin (Mstn) and Muscle RING Finger 1 (MuRF1) were evaluated by real-time quantitative PCR in soleus of flown (BF) and ground control mice (BG). Ppia (cyclophilin A) was used as reference to calculate the delta Ct of the selected genes. Graph shows ΔCt ± SEM; ** p < 0,0075 and * p < 0,025. (TIF) [file pone.0169314.s002.tif]
