# Supplementary material for: Gene Expression Profiling in Slow-Type Calf Soleus Muscle of 30 Days Space-Flown Mice
Source: PLoS One. 2017 Jan 11;12(1):e0169314. doi: 10.1371/journal.pone.0169314 (PMC5226721; doi:10.1371/journal.pone.0169314)
Supplement: S1 Table — The differentially regulated genes (BF vs. BG) in soleus meeting FDR < 0.05 and < -2 & > 2 fold change criteria were analysed by DAVID database and the complete list of genes (part 1) linked to the main functional clusters is included in this table. (PDF) [file pone.0169314.s003.pdf]

S1 Table			SOL						EDL					
			BF vs. BG		FC vs. BG		BF vs. FC		BF vs. BG		FC vs. BG		BF vs. FC	
	Entrez Gene	Gene Symbol	p-value	FC	p-value	FC	p-value	FC	p-value	FC	p-value	FC	p-value	FC
**contractile fiber**	53318	**Pdlim3**	6,13E-05	**2,43**	0,15359	**-1,25**	6,93E-06	**3,04**	0,35996	**1,15**	0,713582	**1,06**	0,575271	**1,09**
	11474	**Actn3**	7,48E-06	**21,58**	0,20284	**1,74**	5,12E-05	**12,41**	0,98737	**-1,01**	0,945965	**1,03**	0,933378	**-1,04**
	107765	**Ankrd1**	0,00088	**9,26**	0,2145	**-1,94**	9,90E-05	**18,00**	0,58023	**1,33**	0,089267	**-2,55**	0,032483	**3,41**
	12569	**Cdk5r1**	0,00934	**2,91**	0,94771	**1,02**	0,01058	**2,85**	0,77095	**-1,11**	0,868538	**-1,06**	0,899725	**-1,05**
	68794	**Flnc**	0,00286	**2,22**	0,48653	**1,17**	0,01079	**1,91**	0,62292	**-1,11**	0,543818	**-1,14**	0,906402	**1,03**
	14201	**Fhl3**	0,00104	**2,10**	0,30586	**1,20**	0,00726	**1,75**	0,65072	**-1,08**	0,73574	**-1,06**	0,907335	**-1,02**
	26556	**Homer1**	0,00026	**2,02**	0,4549	**1,11**	0,00096	**1,81**	0,63115	**1,07**	0,51465	**-1,10**	0,267005	**1,17**
	26557	**Homer2**	2,8E-06	**-3,60**	0,49608	**-1,12**	7E-06	**-3,22**	0,5115	**-1,11**	0,244835	**1,21**	0,081796	**-1,34**
	16669	**Krt19**	0,00021	**2,33**	0,28269	**1,20**	0,00144	**1,94**	0,68203	**1,07**	0,321632	**-1,18**	0,171709	**1,26**
	17883	**Myh3**	3,21E-05	**-5,30**	0,3028	**-1,32**	0,00017	**-4,01**	0,09618	**1,60**	0,408565	**-1,25**	0,020731	**1,99**
	17884	**Myh4**	0,0007	**19,20**	0,79069	**1,19**	0,00113	**16,08**	0,93138	**1,06**	0,87741	**1,11**	0,945627	**-1,05**
	17888 /// 140781	**Myh6 /// Myh7**	8,16E-08	**-4,08**	3,23E-08	**-4,61**	0,3397	**1,13**	0,60809	**1,07**	0,784786	**1,03**	0,80887	**1,03**
	22330	**Vcl**	2,80E-05	**2,05**	0,6087	**1,06**	6,16E-05	**1,93**	0,05416	**1,26**	0,021818	**1,33**	0,626232	**-1,06**
**glucose metabolic process**	72141	**Adpgk**	4,17E-05	**3,97**	0,88647	**1,03**	5,20E-05	**3,84**	0,90908	**1,03**	0,662455	**1,10**	0,746421	**-1,08**
	14120	**Fbp2**	0,00035	**4,15**	0,33063	**1,34**	0,00204	**3,10**	0,60133	**-1,17**	0,906368	**1,04**	0,523732	**-1,21**
	14571	**Gpd2**	6,58E-05	**2,23**	0,0526	**1,33**	0,00247	**1,67**	0,30017	**-1,16**	0,296851	**1,16**	0,050483	**-1,34**
	14555	**Gpd1**	0,00021	**3,97**	0,12162	**1,55**	0,00381	**2,56**	0,12134	**-1,55**	0,658585	**1,13**	0,05551	**-1,75**
	16832	**Ldhb**	0,0048	**-2,01**	0,88792	**1,03**	0,00369	**-2,07**	0,98917	**-1,00**	0,37923	**-1,20**	0,386272	**1,20**
	16846	**Lep**	4,93E-05	**7,78**	0,68591	**1,15**	9,33E-05	**6,78**	0,06066	**1,99**	0,469067	**1,28**	0,210622	**1,55**
	17869	**Myc**	0,00014	**4,70**	0,77277	**-1,09**	8,59E-05	**5,11**	0,84628	**1,06**	0,85636	**1,05**	0,989716	**1,00**
	18682	**Phkg1**	0,00063	**2,44**	0,60886	**-1,11**	0,00026	**2,71**	0,68981	**-1,08**	0,799452	**1,05**	0,516363	**-1,14**
	18597	**Pdha1**	0,00022	**-2,09**	0,00031	**-2,03**	0,83915	**-1,03**	0,96192	**-1,01**	0,002611	**-1,71**	0,002854	**1,70**
	228026	**Pdk1**	1,38E-05	**-4,57**	0,09407	**-1,48**	0,00022	**-3,08**	0,46799	**1,18**	0,023881	**1,75**	0,091339	**-1,49**
	14385	**Slc37a4**	0,00479	**2,87**	0,56022	**1,20**	0,01456	**2,39**	0,51397	**-1,23**	0,867205	**-1,05**	0,624932	**-1,17**
**fatty acid metabolism**	113868	**Acaa1a**	0,00222	**-2,11**	0,49962	**1,14**	0,00065	**-2,41**	0,55026	**1,13**	0,026848	**1,63**	0,080798	**-1,44**
	52538	**Acaa2**	1,27E-05	**-2,68**	0,79535	**1,04**	8,86E-06	**-2,78**	0,04804	**-1,36**	0,437278	**-1,12**	0,187542	**-1,21**
	14081	**Acsl1**	1,21E-05	**-2,00**	7,59E-05	**-1,77**	0,23473	**-1,13**	0,25859	**-1,12**	0,010132	**-1,35**	0,08733	**1,20**
	11430	**Acox1**	3,76E-06	**-2,03**	0,02807	**-1,25**	0,00014	**-1,63**	0,62859	**1,04**	0,02749	**-1,25**	0,01097	**1,30**
	72674	**Adipor1**	0,00028	**2,14**	0,42648	**1,13**	0,00115	**1,89**	0,50795	**1,11**	0,607054	**1,08**	0,879952	**1,02**
	12896	**Cpt2**	5,19E-05	**-2,32**	0,19581	**1,21**	7,35E-06	**-2,81**	0,68653	**-1,06**	0,479292	**1,11**	0,275055	**-1,17**
	231086	**Hadhb**	9,38E-05	**-2,10**	0,32035	**-1,14**	0,00052	**-1,83**	0,23475	**-1,18**	0,381471	**-1,12**	0,737886	**-1,05**
	15446	**Hpgd**	0,00015	**2,59**	0,25324	**-1,23**	2,34E-05	**3,20**	0,74952	**1,06**	0,466377	**-1,14**	0,301798	**1,21**
	15488	**Hsd17b4**	0,00073	**-2,38**	0,07076	**-1,47**	0,0272	**-1,62**	0,02154	**1,66**	0,412507	**1,18**	0,098385	**1,41**
	231086	**Hadhb**	9,38E-05	**-2,10**	0,32035	**-1,14**	0,00052	**-1,83**	0,23475	**-1,18**	0,381471	**-1,12**	0,737886	**-1,05**
**regulation of lipid metabolic process**	11450	**Adipoq**	0,00051	**5,05**	0,05374	**2,09**	0,02464	**2,42**	0,00271	**3,65**	0,000696	**4,74**	0,459882	**-1,30**
	11606	**Agt**	3,10E-06	**3,68**	0,59576	**-1,09**	1,58E-06	**4,02**	0,2658	**1,21**	0,41027	**1,15**	0,758884	**1,05**
	11816	**Apoe**	0,00132	**2,34**	0,0125	**1,82**	0,24337	**1,28**	0,92929	**1,02**	0,928037	**1,02**	0,998746	**-1,00**
	72999	**Insig2**	0,00068	**-2,19**	0,01266	**-1,66**	0,1338	**-1,32**	0,9579	**1,01**	0,151261	**1,30**	0,164938	**-1,29**
	20411	**Sorbs1**	0,00888	**-2,44**	0,2651	**-1,40**	0,07503	**-1,75**	0,02916	**2,03**	0,096792	**1,67**	0,512651	**1,21**
**inflammatory response**	12505	**Cd44**	0,00397	**2,23**	0,8569	**-1,04**	0,00283	**2,32**	0,61562	**-1,12**	0,353551	**-1,24**	0,660968	**1,11**
	20293	**Ccl12**	0,00039	**6,42**	0,72736	**1,15**	0,00071	**5,60**	0,56139	**1,26**	0,737582	**1,14**	0,803498	**1,10**
	20296	**Ccl2**	0,00136	**5,14**	0,57028	**-1,26**	0,00049	**6,48**	0,6249	**-1,22**	0,68152	**-1,18**	0,936598	**-1,03**
	20306	**Ccl7**	0,00041	**2,79**	0,69608	**-1,09**	0,00021	**3,04**	0,4399	**1,19**	0,902712	**1,03**	0,513085	**1,15**
	14825	**Cxcl1**	0,00821	**2,88**	0,99689	**1,00**	0,00827	**2,88**	0,96802	**1,01**	0,765533	**-1,11**	0,735327	**1,12**
	15945	**Cxcl10**	0,00273	**2,11**	0,80857	**-1,05**	0,00174	**2,22**	0,20113	**-1,31**	0,188058	**-1,32**	0,966223	**1,01**
	55985	**Cxcl13**	0,00142	**2,22**	0,97202	**-1,01**	0,00133	**2,24**	0,98623	**1,00**	0,740463	**-1,07**	0,727557	**1,07**
	12266	**C3**	0,0053	**2,14**	0,00301	**2,29**	0,76418	**-1,07**	0,38387	**-1,22**	0,119109	**1,45**	0,024001	**-1,78**
	11537	**Cfd**	0,00398	**3,13**	0,04125	**2,08**	0,22928	**1,50**	0,01778	**2,42**	0,002263	**3,46**	0,286007	**-1,43**
	13850	**Ephx2**	0,0002	**-2,24**	0,74361	**1,05**	0,00012	**-2,36**	0,29192	**-1,18**	0,551147	**1,10**	0,111911	**-1,30**
	80859	**Nfkbiz**	0,00273	**2,48**	0,04768	**1,70**	0,14654	**1,45**	0,77668	**-1,07**	0,586718	**-1,14**	0,792904	**1,07**
	18405	**Orm1**	7,98E-05	**5,59**	0,64995	**1,15**	0,00017	**4,88**	0,01905	**2,22**	0,334438	**1,35**	0,114579	**1,65**
	20698	**Sphk1**	0,00235	**4,13**	0,67977	**-1,17**	0,0011	**4,83**	0,81777	**-1,09**	0,819476	**-1,09**	0,998237	**-1,00**
	20818 /// 22041	**Srprb /// Trf**	0,00112	**2,54**	0,05021	**1,61**	0,06	**1,58**	0,55607	**1,14**	0,043247	**1,64**	0,12404	**-1,44**
	22361	**Vnn1**	5,59E-05	**2,79**	0,13121	**1,32**	0,00079	**2,12**	0,23284	**1,24**	0,846964	**-1,03**	0,171674	**1,28**
