# Supplementary material for: Gene Expression Profiling in Slow-Type Calf Soleus Muscle of 30 Days Space-Flown Mice
Source: PLoS One. 2017 Jan 11;12(1):e0169314. doi: 10.1371/journal.pone.0169314 (PMC5226721; doi:10.1371/journal.pone.0169314)
Supplement: S2 Table — The differentially regulated genes (BF vs. BG) in soleus meeting FDR < 0.05 and < -2 & > 2 fold change criteria were analysed by DAVID database and the complete list of genes (part 2) linked to the main functional clusters is included in this table. (PDF) [file pone.0169314.s004.pdf]

S2 Table

			SOL						EDL					
			BF vs. BG		FC vs. BG		BF vs. FC		BF vs. BG		FC vs. BG		BF vs. FC	
	Entrez Gene	Gene Symbol	p-value	FC	p-value	FC	p-value	FC	p-value	FC	p-value	FC	p-value	FC
**cellular calcium ion homeostasis**	20203	**S100b**	0,00346	**2,75**	0,30042	**1,35**	0,02566	**2,03**	0,97978	**1,01**	0,855268	**-1,05**	0,83548	**1,06**
	14062	**F2r**	0,00131	**-2,18**	0,17305	**1,31**	0,00011	**-2,85**	0,26237	**-1,25**	0,718653	**1,07**	0,148315	**-1,33**
	16438	**Itpr1**	0,00547	**2,63**	0,12286	**1,61**	0,11101	**1,64**	0,49471	**1,22**	0,159926	**1,53**	0,442663	**-1,25**
	18821	**Pln**	1,26E-10	**-3,47**	4,31E-07	**-1,83**	2,47E-07	**-1,89**	0,10252	**1,12**	0,000192	**-1,39**	1,33E-05	**1,55**
	19218	**Ptger3**	4,44E-06	**6,91**	0,0173	**1,97**	0,00026	**3,51**	0,06721	**1,64**	0,341052	**1,28**	0,327545	**1,28**
	18750	**Prkca**	7,98E-06	**2,01**	0,18989	**1,14**	5,87E-05	**1,76**	0,01436	**1,31**	0,402483	**1,09**	0,069658	**1,21**
**stress response**	81489	**Dnajb1**	0,00147	**10,16**	0,68665	**-1,26**	0,00071	**12,84**	0,79053	**-1,17**	0,579058	**-1,38**	0,770364	**1,18**
	15507	**Hspb1**	9,29E-05	**2,53**	0,56781	**1,10**	0,00024	**2,30**	0,36546	**1,16**	0,009995	**-1,64**	0,001776	**1,91**
	56534	**Hspb3**	0,00492	**2,02**	0,12824	**-1,40**	0,00027	**2,82**	0,71816	**1,08**	0,821258	**-1,05**	0,559362	**1,13**
	80888	**Hspb8**	0,00301	**2,45**	0,00129	**-2,75**	4,38E-06	**6,73**	0,97127	**1,01**	0,103772	**-1,53**	0,097494	**1,54**
	269951	**Idh2**	2,49E-07	**-3,53**	0,14739	**-1,21**	1,41E-06	**-2,92**	0,04456	**1,31**	0,822024	**-1,03**	0,029338	**1,35**
	15519	**Hsp90aa1**	0,00588	**3,22**	0,06205	**-2,05**	0,00016	**6,62**	0,91835	**1,04**	0,166326	**-1,67**	0,140478	**1,74**
	17872	**Ppp1r15a**	0,00217	**2,61**	0,35469	**-1,27**	0,0004	**3,31**	0,67778	**-1,11**	0,181196	**-1,42**	0,339973	**1,28**
**regulation of muscle contraction**	11938	**Atp2a2**	0,00035	**-2,74**	0,77862	**1,06**	0,00022	**-2,91**	0,50012	**-1,15**	0,840265	**1,04**	0,385186	**-1,20**
	11928	**Atp1a1**	0,00184	**-2,18**	0,09073	**-1,43**	0,05398	**-1,52**	0,32868	**-1,22**	0,858518	**1,04**	0,253174	**-1,27**
	98660	**Atp1a2**	4,68E-06	**-2,17**	0,76078	**-1,03**	7,06E-06	**-2,10**	0,00015	**1,71**	0,535701	**-1,06**	5,55E-05	**1,82**
	11931	**Atp1b1**	2,80E-03	**-2,04**	0,29087	**-1,23**	2,16E-02	**-1,65**	0,83543	**-1,04**	0,804392	**-1,05**	9,68E-01	**1,01**
	11932	**Atp1b2**	4,69E-04	**3,68**	0,03609	**1,91**	3,38E-02	**1,93**	0,45275	**-1,24**	0,402922	**1,27**	1,26E-01	**-1,57**
	12373	**Casq2**	1,82E-07	**-4,16**	0,92487	**-1,01**	2,01E-07	**-4,11**	0,69434	**-1,06**	0,475631	**-1,10**	0,744269	**1,05**
	16531	**Kcnma1**	2,70E-07	**-3,83**	0,96532	**-1,01**	2,83E-07	**-3,81**	0,18298	**-1,20**	0,01542	**1,45**	0,001158	**-1,74**
**muscle organ development**	112405	**Egln1**	6,89E-06	**-2,26**	0,1791	**-1,17**	5,26E-05	**-1,94**	0,88569	**1,02**	0,566969	**-1,07**	0,47613	**1,08**
	12803 /// 109910 /// 664779	**Cntf /// Zfp91 /// Zfp91Cntf**	0,00071	**3,96**	0,96517	**-1,01**	0,00066	**4,02**	0,2215	**1,48**	0,430493	**-1,28**	0,056995	**1,90**
	14062	**F2r**	0,00131	**-2,18**	0,17305	**1,31**	0,00011	**-2,85**	0,26237	**-1,25**	0,718653	**1,07**	0,148315	**-1,33**
	12814	**Col11a1**	0,00532	**-2,75**	0,4654	**-1,25**	0,02154	**-2,19**	0,12119	**1,64**	0,011364	**2,43**	0,212171	**-1,48**
	18198	**Musk**	0,00407	**3,16**	0,54722	**-1,22**	0,00132	**3,87**	0,08774	**1,83**	0,396527	**1,33**	0,346645	**1,38**
	17927	**Myod1**	0,00432	**4,72**	0,3483	**1,54**	0,02633	**3,06**	0,45904	**-1,40**	0,981518	**1,01**	0,445604	**-1,42**
	17878	**Myf6**	4,25E-05	**2,58**	0,0477	**-1,40**	2,12E-06	**3,60**	0,00053	**2,03**	0,378428	**-1,15**	0,000117	**2,33**
	17928	**Myog**	7,27E-05	**3,52**	0,17977	**1,36**	0,00076	**2,60**	0,87107	**1,04**	0,818722	**-1,05**	0,696128	**1,09**
	71371	**Arid5b**	0,00325	**2,85**	0,37016	**1,30**	0,01819	**2,18**	0,22658	**1,44**	0,533446	**1,20**	0,538364	**1,20**
**response to oxidative stress**	18104	**Nqo1**	0,00156	**-2,12**	0,40971	**-1,17**	0,00744	**-1,81**	0,61575	**1,10**	0,297234	**1,22**	0,576249	**-1,11**
	12359	**Cat**	0,00235	**-2,48**	0,15179	**-1,44**	0,03943	**-1,73**	0,32971	**1,27**	0,061393	**1,63**	0,315489	**-1,28**
	69590	**Gpx8**	0,00142	**-2,18**	0,00769	**-1,83**	0,37455	**-1,19**	0,20975	**1,28**	0,841258	**-1,04**	0,151944	**1,34**
	67092	**Gatm**	0,00731	**2,35**	0,44299	**1,23**	0,03173	**1,91**	0,52839	**1,19**	0,85534	**1,05**	0,651643	**1,13**
	18477 /// 100862012	**Gm21399 /// Prdx1**	0,00776	**-2,13**	0,01998	**-1,89**	0,61958	**-1,13**	0,06797	**1,61**	0,135117	**1,46**	0,69356	**1,10**
	76650	**Srxn1**	0,00406	**3,25**	0,06409	**-1,97**	0,00012	**6,41**	0,6365	**-1,18**	0,17442	**-1,62**	0,35657	**1,38**
