# Supplementary material for: Gene Expression Profiling in Slow-Type Calf Soleus Muscle of 30 Days Space-Flown Mice
Source: PLoS One. 2017 Jan 11;12(1):e0169314. doi: 10.1371/journal.pone.0169314 (PMC5226721; doi:10.1371/journal.pone.0169314)
Supplement: S3 Table — The differentially regulated genes (BF vs. BG) in soleus meeting FDR < 0.05 and < -2 & > 2 fold change criteria were analysed by DAVID database and the complete list of genes (part 3) linked to the main functional clusters is included in this table. (PDF) [file pone.0169314.s005.pdf]

3S Table			SOL						EDL					
			BF vs. BG		FC vs. BG		BF vs. FC		BF vs. BG		FC vs. BG		BF vs. FC	
	Entrez Gene	Gene Symbol	p-value	FC	p-value	FC	p-value	FC	p-value	FC	p-value	FC	p-value	FC
**regulation of programmed cell death**	229595	**Adamtsl4**	8,08E-05	**-2,29**	0,02751	**1,43**	2,45E-06	**-3,27**	0,90737	**1,02**	0,953995	**1,01**	0,9532	**1,01**
	12227	**Btg2**	0,0003	**3,29**	0,95515	**-1,01**	0,00027	**3,34**	0,11337	**1,50**	0,416429	**-1,22**	0,025485	**1,83**
	12125	**Bcl2l11**	1,75E-05	**2,08**	0,32317	**1,12**	8,11E-05	**1,86**	0,96995	**1,00**	0,376675	**-1,10**	0,357685	**1,11**
	13347	**Dffa**	1,45E-09	**-3,28**	0,00016	**-1,48**	1,33E-07	**-2,22**	0,07498	**-1,15**	0,695083	**1,03**	0,036638	**-1,19**
	21847	**Klf10**	0,00136	**2,50**	0,63749	**1,11**	0,00327	**2,24**	0,60502	**-1,12**	0,06859	**-1,56**	0,167447	**1,38**
	12421	**Rb1cc1**	0,00729	**-2,07**	0,1654	**-1,40**	0,106	**-1,49**	0,12999	**1,44**	0,079172	**1,54**	0,774752	**-1,07**
	12803 /// 109910 /// 664779	**Cntf /// Zfp91 /// Zfp91Cntf**	0,00071	**3,96**	0,96517	**-1,01**	0,00066	**4,02**	0,2215	**1,48**	0,430493	**-1,28**	0,056995	**1,90**
	14311	**Cidec**	0,00072	**5,58**	0,24753	**1,59**	0,0065	**3,51**	0,16671	**1,75**	0,063929	**2,18**	0,580255	**-1,24**
	12569	**Cdk5r1**	0,00934	**2,91**	0,94771	**1,02**	0,01058	**2,85**	0,77095	**-1,11**	0,868538	**-1,06**	0,899725	**-1,05**
	12575	**Cdkn1a**	0,0035	**2,61**	0,00424	**-2,54**	1,18E-05	**6,61**	0,00178	**2,88**	0,02099	**-2,02**	2,37E-05	**5,81**
	109006	**Ciapin1**	0,00148	**-2,12**	0,48828	**-1,14**	0,00545	**-1,86**	0,47257	**-1,15**	0,906883	**-1,02**	0,545467	**-1,12**
	71228	**Dlg5**	0,00932	**2,14**	0,75447	**1,08**	0,01689	**1,98**	0,9862	**-1,00**	0,630518	**1,13**	0,61846	**-1,13**
	14573	**Gdnf**	0,00491	**4,76**	0,93084	**1,04**	0,00578	**4,57**	0,74974	**1,16**	0,46315	**-1,41**	0,299545	**1,64**
	16443	**Itsn1**	0,0082	**-2,81**	0,13177	**-1,70**	0,14856	**-1,66**	0,51139	**1,25**	0,264776	**1,47**	0,630858	**-1,17**
	17153	**Mal**	0,0039	**2,24**	0,11032	**1,48**	0,09084	**1,51**	0,99883	**1,00**	0,612508	**-1,12**	0,611497	**1,12**
	17869	**Myc**	0,00014	**4,70**	0,77277	**-1,09**	8,59E-05	**5,11**	0,84628	**1,06**	0,85636	**1,05**	0,989716	**1,00**
	18049	**Ngf**	0,00456	**2,11**	0,76654	**1,07**	0,008	**1,98**	0,85196	**-1,04**	0,945216	**-1,02**	0,906069	**-1,03**
	18477 /// 100862012	**Gm21399 /// Prdx1**	0,00776	**-2,13**	0,01998	**-1,89**	0,61958	**-1,13**	0,06797	**1,61**	0,135117	**1,46**	0,69356	**1,10**
	18712	**Pim1**	0,00546	**2,19**	0,6161	**-1,13**	0,00213	**2,47**	0,69216	**-1,10**	0,109063	**-1,50**	0,209738	**1,36**
	20698	**Sphk1**	0,00235	**4,13**	0,67977	**-1,17**	0,0011	**4,83**	0,81777	**-1,09**	0,819476	**-1,09**	0,998237	**-1,00**
	22361	**Vnn1**	5,59E-05	**2,79**	0,13121	**1,32**	0,00079	**2,12**	0,23284	**1,24**	0,846964	**-1,03**	0,171674	**1,28**
**Focal adhesion**	12845	**Comp**	0,00846	**-2,91**	0,20312	**1,58**	0,00074	**-4,60**	0,53184	**-1,24**	0,074451	**1,94**	0,023336	**-2,42**
	12643	**Chad**	0,00414	**-4,87**	0,52277	**1,34**	0,00125	**-6,54**	0,21887	**-1,79**	0,047454	**2,69**	0,004338	**-4,82**
	12842	**Col1a1**	0,00742	**-3,68**	0,19247	**1,75**	0,00061	**-6,43**	0,64599	**1,21**	0,065843	**2,27**	0,146468	**-1,87**
	12825	**Col3a1**	0,0034	**-2,83**	0,60482	**1,16**	0,0013	**-3,30**	0,46028	**1,24**	0,274835	**1,39**	0,70959	**-1,12**
	12814	**Col11a1**	0,00532	**-2,75**	0,4654	**-1,25**	0,02154	**-2,19**	0,12119	**1,64**	0,011364	**2,43**	0,212171	**-1,48**
	16782	**Lamc2**	0,00279	**2,02**	0,71827	**-1,07**	0,00143	**2,16**	0,89053	**1,03**	0,818956	**-1,04**	0,71453	**1,07**
	228785	**Mylk2**	0,00749	**2,21**	0,2999	**1,31**	0,05491	**1,69**	0,54211	**-1,17**	0,625517	**-1,13**	0,901369	**-1,03**
	18479	**Pak1**	0,00589	**2,37**	0,68338	**1,11**	0,01279	**2,13**	0,30309	**-1,32**	0,964635	**1,01**	0,284109	**-1,34**
	21894	**Tln1**	0,00011	**2,45**	0,29812	**-1,19**	2,15E-05	**2,91**	0,61681	**-1,09**	0,56217	**-1,10**	0,935643	**1,01**
	22340	**Vegfb**	4,66E-05	**-2,13**	0,45475	**1,10**	1,51E-05	**-2,34**	0,00307	**-1,57**	0,302338	**-1,14**	0,022582	**-1,38**
**Other/unknown**	19017	**Ppargc1a**	0,00267	**-2,55**	0,05259	**-1,70**	0,13144	**-1,49**	0,39786	**-1,24**	0,56415	**1,16**	0,167349	**-1,44**
	170826	**Ppargc1b**	0,00023	**-2,22**	0,35479	**1,16**	5,07E-05	**-2,57**	0,12471	**-1,29**	0,771826	**-1,05**	0,200667	**-1,23**
	19013	**Ppara**	2,75E-05	**-4,18**	0,72112	**1,08**	1,63E-05	**-4,53**	0,0683	**-1,55**	0,874348	**-1,04**	0,090404	**-1,50**
	19082	**Prkag1**	7,39E-05	**-2,38**	0,83774	**-1,03**	0,0001	**-2,31**	0,46806	**1,12**	0,251158	**1,19**	0,656297	**-1,07**
	108099	**Prkag2**	0,00096	**2,36**	0,34429	**1,21**	0,00571	**1,94**	0,8476	**-1,04**	0,300454	**1,24**	0,225237	**-1,29**
	14371	**Fzd9**	2,21E-10	**-6,75**	0,91788	**1,01**	2,07E-10	**-6,82**	0,00555	**-1,40**	0,122325	**1,18**	0,000292	**-1,65**
	211949	**Spsb4**	8,95E-06	**-4,26**	0,67818	**-1,09**	1,61E-05	**-3,92**	0,88774	**1,03**	0,587332	**1,12**	0,686562	**-1,09**
	14367	**Fzd5**	0,00144	**2,72**	0,61719	**1,13**	0,00365	**2,40**	0,57744	**-1,15**	0,499997	**-1,18**	0,904285	**1,03**
	13653	**Egr1**	0,00513	**5,58**	0,32053	**-1,68**	0,00079	**9,40**	0,3657	**1,61**	0,758168	**1,17**	0,543602	**1,37**
	59011	**Myoz1**	0,00011	**5,04**	0,55285	**1,19**	0,00029	**4,23**	0,09327	**1,69**	0,671605	**1,13**	0,19018	**1,49**
