# Supplementary material for: Gene Expression Profiling in Slow-Type Calf Soleus Muscle of 30 Days Space-Flown Mice
Source: PLoS One. 2017 Jan 11;12(1):e0169314. doi: 10.1371/journal.pone.0169314 (PMC5226721; doi:10.1371/journal.pone.0169314)
Supplement: S4 Table — HK, Reference genes; bp, expected product size; T°, annealing temperature. * Sandonà D. et al. (2012) Adaptation of Mouse Skeletal Muscle to Long-Term Microgravity in the MDS Mission. PLoS ONE 7(3): e33232. (PDF) [file pone.0169314.s006.pdf]

S4 Table

**Protein**	Genes	Sequence NCBI	Forward primer 5' → 3'	Reverse primer 3' → 5'	bp	T°
**HK GENES**						
β-actin*	*Actb*	NM_007393.5	CAAACATCCCCCAAAGTTCTAC	TGAGGGACTTCCTGTAACCACT	135	57,7
cyclophilin A *	*Ppia*	NM_008907.1	AGCATGTGGTCTTTGGGAAGGTG	CTTCTTGCTGGTCTTGCCATTCC	92	57,7
GAPDH *	*Gapdh*	NM_001289726.1	TGTGTCCGTCGTGGATCTGA	TTGCTGTTGAAGTCGCAGGAG	150	57,7
**SPECIFIC GENES**						
Calsequestrin 2	*Casq2*	NM_009814	TCACCAGTGGTAAGAGAGGCT	ACTCTTAATGGTCGCTGCCC	238	59
peroxisome proliferator activated receptor alpha	*Ppara*	NM_011144.6	GACAGTGACAGACAACGGCA	GTGGCAGGAAGGGAACAGAC	144	59
actinin 3	*Acnt3*	NM_013456.2	CCAGGAGCAGCTCAACGAAT	TTGTTCAGCCGTGTCTGTCT	238	57,7
myogenic factor 6	*Myf6*	NM_008657.2	TGCTAAGGAAGGAGGAGCAA	CCTGCTGGGTGAAGAATGTT	166	57,7
frizzled class receptor 9	*Fzd9*	NM_010246.1	TTATGGTTGCTCCCTCCTTG	CACTCCCTGCATGAGACAGA	162	59
K+ large conductance Ca++-act channel, subfamily M, alpha 1	Kcnma1	NM_001253358.1	GTCAGCGTCCTGGACTCAC	ATGTCTGCGGAGTGCTGTAG	140	59
myostatin	*Mstn*	NM_010834.3	TCACGCTACCACGGAAACAA	AGGAGTCTTGACGGGTCTGA	166	57,7
MuRF-1 E3 ligase *	*Trim63*	NM_001039048.2	ACCTGCTGGTGGAAAACATC	CTTCGTGTTCCTTGCACATC	96	57,7
